# C-Terminal Domain of Hemocyanin, a Major Antimicrobial Protein from *Litopenaeus vannamei*: Structural Homology with Immunoglobulins and Molecular Diversity

**DOI:** 10.3389/fimmu.2017.00611

**Published:** 2017-06-13

**Authors:** Yue-Ling Zhang, Bo Peng, Hui Li, Fang Yan, Hong-Kai Wu, Xian-Liang Zhao, Xiang-Min Lin, Shao-Ying Min, Yuan-Yuan Gao, San-Ying Wang, Yuan-You Li, Xuan-Xian Peng

**Affiliations:** ^1^Department of Biology and Guangdong Provincial Key Laboratory of Marine Biotechnology, School of Sciences, Shantou University, Shantou, China; ^2^Center for Proteomics, State Key Laboratory of Biocontrol, School of Life Sciences, Sun Yat-sen University, University City, Guangzhou, China; ^3^School of Life Sciences, Xiamen University, Xiamen, Fujian, China

**Keywords:** hemocyanin, diversity, defense mechanisms, immunoglobulins, shrimp

## Abstract

Invertebrates rely heavily on immune-like molecules with highly diversified variability so as to counteract infections. However, the mechanisms and the relationship between this variability and functionalities are not well understood. Here, we showed that the C-terminal domain of hemocyanin (HMC) from shrimp *Litopenaeus vannamei* contained an evolutionary conserved domain with highly variable genetic sequence, which is structurally homologous to immunoglobulin (Ig). This domain is responsible for recognizing and binding to bacteria or red blood cells, initiating agglutination and hemolysis. Furthermore, when HMC is separated into three fractions using anti-human IgM, IgG, or IgA, the subpopulation, which reacted with anti-human IgM (HMC-M), showed the most significant antimicrobial activity. The high potency of HMC-M is a consequence of glycosylation, as it contains high abundance of α-d-mannose relative to α-d-glucose and *N*-acetyl-d-galactosamine. Thus, the removal of these glycans abolished the antimicrobial activity of HMC-M. Our results present a comprehensive investigation of the role of HMC in fighting against infections through genetic variability and epigenetic modification.

## Introduction

One of the major goals of comparative immunobiology is to identify molecules, which function in non-self recognition in invertebrates (1). Invertebrates do not possess lymphocytes or antibody-based humoral immune response that plays a vital role in vertebrate immunity, yet they nonetheless are capable of mounting effective immune responses, called innate immunity (2, 3). Unlike adaptive immunity, which is only found in vertebrates, innate immunity is found in all animals with and without adaptive immunity. The innate immune system relies on its capability to rapidly detect pathogen-associated molecular patterns (PAMPs) of invading pathogenic microbes as foreign intruders for elimination (4–6). This detection system is mainly based on pattern recognition receptors (PRRs). Recently, several families of PRRs have been described, including the Toll-like receptors (TLRs) (7, 8), the mammalian NOD-like receptors (NLRs) (9), RIG-1-like RNA helicases (RLHs) (10), pyrin and HIN domain-containing protein (PYHIN) family members (11), plant nucleotide-binding site-leucine-rich repeat (NBS-LRR) proteins (12), C-type lectin receptors (CLRs) (13, 14), and “triggering receptor expressed on myeloid cells” (TREM) proteins (15, 16). TLRs, which are classical transmembrane proteins, recognize either extracellular or membrane-encased foreign organisms (7), while NLRs and RLHs are cytoplasmic molecules, which sense intracellular invaders (9, 10). Similarly, plant NBS-LRRs respond either directly or indirectly to pathogens (12), while Myeloid CLRs, including the cell surface molecules DC-SIGN and dectin-1, promote synergistic immune responses with other innate receptors and/or probably bind host-derived molecules (13). For TREM proteins, these respond to both endogenous and exogenous danger signals and function as “amplifiers” of innate responses and as regulators of developmental stage- and tissue-specific functions (16).

Natural antibodies and complement are two major plasma proteins, which are a relatively conserved frontline non-TLR members in the circulatory system of vertebrates (17). While invertebrates lack immunoglobulins (Igs) and the complement system, they have an array of frontline defense molecules (18). These defense molecules seem to have two characteristics, i.e., they are homologs with the immune defense molecules of higher vertebrates, and the diversity of the molecules does not appear to closely relate to the molecules of the adaptive system (3, 18, 19). Recently, the ancient origin of the complement system was reported in the horseshoe crab, an ancient protostome that is dubbed a “living fossil” (18). Molecular diversity of PRRs has also been found in invertebrates. Reports indicate that the IgSF1 domain of the fibrinogen-related protein 3 (FREP3) subfamily is diversified with molecules involved in internal defense of snails (3). Down syndrome cell adhesion molecule (Dscam), which consist of more than 18,000 isoforms of the Ig-superfamily receptor, has been detected in the hemolymph and is reported to be required in hemocytes for efficient phagocytosis and binding to *Escherichia coli* in *Drosophila* (19). These studies may hold some clues in the discovery of frontline non-TLR defense mechanisms in invertebrates, as well as the origin of immune molecules including Ig and complement components in these organisms.

Hemocyanin (HMC), a respiratory protein, is a major glycoprotein in Arthropoda and Mollusca, accounting for approximately 90% of their plasma proteins. Apart from its canonical role, HMC can function in the frontline immune protection in crustaceans (18). A number of studies have shown that HMC is functionally converted into phenoloxidase-like enzyme with or without proteolytic cleavage and therefore contributes to the antibacterial activity (20–23). Besides this, HMC exerts a non-specific antiviral activity with no adverse cytotoxic effect to the host cells (24). In a shrimp subtractive library, the HMC gene was found to account for 66.25% or 265 out of a total 400 clones in WSSV-resistant shrimps (25). Moreover, our previous research indicated that the HMC of *Litopenaeus vannamei* or *Scylla serrata* could directly bind with several pathogens and animal erythrocytes, suggesting that it possessed agglutinative and hemolytic activities (26–28). These results have helped to uncover the antimicrobial action of HMC through conversion to phenoloxidase-like enzymes and peptide fragments. However, there is currently limited information on the gene evolution of HMC, how it recognizes PAMPs, as well as molecular diversity and involvement in immunosurveillance. Thus, there is the need to further examine the antibacterial mechanisms of this protein so as to give us a better insight into its role in immunity.

Here, we report on the use of bacterial pull-down and proteomic techniques to identify HMC as a major PRR in shrimp followed by the use of Far-Western blot analysis to characterize PAMPs recognizable by this PRR. The immune defense ability of this protein was also characterized. Our findings revealed that the C-terminal domain (Ig-like domain, D3) of HMC probably through convergent evolution is able to provide HMC the ability to recognize the outer membrane (OM) of several bacterial proteins. More importantly, HMC was shown to be widely diversed in its response and reactivity to heterogeneous antibodies, bacterial agglutination, inhibition of bacterial growth, and hemolytic activity toward human erythrocytes. Our study therefore reveals HMC as a novel PRR molecule, which has diverse functions, and is probably the Ig homolog in crustacean; a finding which could provide us with further clues into exploring the origin of the various Igs.

## Materials and Methods

### Preparation of Shrimp Hemolymph

Penaeid shrimps *L. vannamei* from natural source, weighing 15–20 g and irrespective of sex, were cultured in aerated seawater. Hemolymph was drawn directly from the pericardial sinus using a sterile needle and syringe, and then allowed to clot overnight at 4°C. Pooled sera was collected after centrifuging at 3,000*g* for 20 min and stored at −20°C until analysis. All animal experiments were carried out in accordance with the guidelines and approval of the Animal Research and Ethics Committees at Sun Yat-sen University, Shantou University, and Xiamen University, respectively.

### Bacterial Strains and Growth Conditions

The bacterial strains used in the current study were *E. coli* K12 99+, *E. coli* K12 BW25113 and its genetically modified strains with gene deletion (Δ*ompT*, Δ*ompA*, Δ*fadL*, Δ*ompW*, Δ*ompX*, Δ*lamB*, and Δ*dps*), *E. coli* DH5α, *E. coli* XL1-Blue MRF, *Vibrio parahaemolyticus, Vibrio alginolyticus, Vibrio harveyi, Vibrio fluvialis, Vibrio anguillarum, Aeromonas hydrophila, Aeromonas sobria, Pseudomonas fluorescens*, and *Staphylococcus aureus*. Of these, *E. coli* K12 BW25113 and its gene-deleted mutants were kindly provided by NBRP (NIG, Japan): *E. coli* (29), while Δ*ompC* and the other bacterial strains are collections in our laboratory. All strains were cultured under standard laboratory procedures. In brief, *E. coli* K12 and *S. aureus* strains were grown at 37°C in Luria Bertani (LB), and the other strains were grown at 28°C with shaking 200 rpm/min in Broth medium. All bacterial cultures were grown in LB or Broth medium from frozen stock in a shaker bath for 16 h. The bacterial cells were diluted into 1:100 using fresh medium and collected at the exponential phase (OD_600_ = 0.6) for further study.

### Characterization of Frontline Immune Proteins in Shrimp Plasma Using Inactivated Bacteria as Affinity Matrix

Four species of bacteria *V. parahaemolyticus, V. fluvialis, P. fluorescens*, and *E. coli* K12 were used as affinity matrix. These bacteria were separately cultured, harvested, washed, and inactivated (*V. parahaemolyticus* and *E. coli* K12, and *V. fluvialis* and *P. fluorescens* were treated, respectively, at 100°C for 5 min, and at 80°C for 10 min). The cells were then incubated with a Tris-HCl buffered saline (1 M, pH 8.0) for 3 h, centrifuged at 5,000*g* for 10 min, washed three times with a saline buffer and resuspended in the saline buffer. 0.5 mL of each bacteria suspension (1 × 10^9^ CFU/mL) was incubated with shrimp sera (1:1) at room temperature for 5 h. After centrifuging at 5,000*g* for 10 min and washing three times with saline buffer, the resulting pellet was resuspended in Tris-HCl buffered saline (1 M, pH 8.0) at room temperature for 2 h. After centrifuging at 12,000*g* for 10 min, the supernatant was concentrated by the method of acetone precipitation and dissolved in a saline buffer. The frontline immune proteins in shrimp sera were then characterized using one-dimensional sodium sulfate-polyacrylamide gel electrophoresis (1-DE), Western blot, mass spectrometry, and agglutination assays.

### Sequence Analysis and Phylogenetic Studies

Matrices of pairwise maximum-likelihood distance estimates were computed from the aligned HMC domain sequences using the Jones-Taylor-Thornton (JTT) substitution matrix (30, 31). Complete nucleotide sequences of *L. vannamei* and closely related species, i.e., *Palinurus vulgaris, Pacifastacus leniusculus, Homarus americanus, Palinurus elephas*, and *Gammarus roeseli* used for the analysis are available from the NCBI mRNA database. Similarly, the NCBI conserved domain database and search service was employed in determining the three domains of HMC (32). The six coding sequences (CDS) were translated into amino acid sequences, and the three domains obtained from each of the six proteins coded by the six CDSs. Alignments were performed based on each of the three domains. The BLOSUM (blocks substitution matrix) scores were derived from local, ungapped alignments of a large number of distantly related proteins (33). Several variations of the BLOSUM matrix are available (e.g., BLOSUM 62) where the number refers to the minimum percentage of identity of the blocks used to construct the matrix. In contrast, the JTT substitution matrix uses a large collection of global alignments of closely related sequences. The accumulation of antonymous (Ka) substitutions was determined by Li’s method. Immune-related domains were downloaded from the NCBI conserved domain database (34). Each row of conserved amino acids and alterable residue numbers (in square brackets) in the domain sequence alignment were defined as a unit. A PERL script was developed to align HMC D3 to these units in turn. The three-dimensional structures were modeled through the NCBI free software Cn3D http://www.ncbi.nlm.nih.gov/Structure/CN3D/cn3d.shtml. For the characterization of the association of HMC D3 with the Ig superfamily molecules, the amino acid sequences of shrimp HMC [*L. vannamei*: S-HMC (gi|854403) and L-HMC (gi|7414468), S-HMC shared 79% amino acid identity and 87% amino acid homology with L-HMC; *P. monodon* HMC (gi|16612121)] and Ig superfamily molecules including human IgG heavy and light chains from the NCBI database were aligned using sequence alignment programs (Clustal X and BioEdit).

### Analysis of SNPs Profile in the HMC D3 Domain

To determine the SNP sites in the S-HMC (X82502.1, 1,741–2,528 bp) and L-HMC (AJ250830.1, 1,258–2,016 bp) D3 domains in the tissues, individuals and developmental stages, PCR-based DNA/cDNA libraries were constructed as previously described with modifications (35). Briefly, DNA was obtained from heart, stomach, gill and hepatopancreas of shrimp A, or hepatopancreas of shrimp B and C, or nauplius larva, using the Genome DNA Extraction Kit (Dongsheng Biotech Company, China). Total RNA was extracted from these samples as described in DNA extraction using RNAiso Plus (Takara, Japan) according to manufacturer instructions. The extracted RNA was treated with RNase-Free DNase (Takara, Japan) to remove contaminating DNA, and cDNA was synthesized using the M-MLV RTase cDNA Synthesis Kit (Takara, Japan) following the manufacturer’s instructions. Total DNA and cDNA were used to perform PCR analysis using S-HMC D3 primers (S-HMC D3-F: 5′-CAAGGACAACCTACCCC-3′ and S-HMC D3-R: 5′-GAAGAGTTGTAAGCTTGTAATC-3′) and L-HMC D3 primers (L-HMC D3-F: 5′-ACAGTGGAAGAACTAACATT-3′ and L-HMC D3-R: 5′-CCCTCGAGTTAATGATGGATATGCT-3′), respectively. The thermal cycling conditions were as follows: 94°C for 5 min, 32 cycles of 94°C for 30 s, 60°C (S-HMC D3) or 52°C (L-HMC D3) for 30 s, 72°C for 60 s, and 72°C for 10 min. The PCR products were extracted, inserted into the pMD-19T vector (Takara, Japan) and then transformed in *E. coli* DH5α (Promega, Madison, WI, USA). Positives colonies were selected on LB agar plates containing 40 mg/mL 5-bromo-4-chloro-3-indoly-L-β-d-galactoside and 100 µg/mL ampicillin (Sangon, Shanghai, China). A total of 28 libraries each containing 10^6^ clones were established. Approximately 96 positive colonies from each library were randomly picked into a 96-well plate and sequenced by Shenzhen Genomics Institute (Shenzhen, China) or Shanghai Sangon Biological Engineering Technology & Services Co., Ltd. (Shanghai, China). The HMC D3 sequences from each library were aligned using Clustal X and BioEdit, and SNPs were identified. A SNP was designated as a nucleotide that had been substituted no less than two times in comparison to S-HMC D3 (X82502.1) or L-HMC D3 (AJ250830.1) domain sequence. All SNP sites were listed and compared by statistical analysis.

### Gene Cloning, Expression and Purification of HMC and Its Fragments

RNA isolation from shrimp *L. vannamei* hepatopancreas and cDNA synthesis was carried out as described in the section on SNP profile analysis. Four pairs of primers, viz. S-HMC primers S-HMC-F (5′-CGGAGCTCAGTTCACCATCAGCACCA-3′) and S-HMC-R (5′-GGCTGCAGGAAGAGTTGTAAGCTTGAATC-3′), S-HMC D3 primers S-HMC-D3-F (5′-CGGAGCTCTACCCCCATACA CCAAAGC-3′) and S-HMC-D3-R (5′-GGCTGCAGGAAGAGTTGTAAGCTTGAATC-3′), S-HMC D1 + D2A primers S-HMC-(D1 + D2A)-F (5′-CGGAGCTCAGTTCACCATCAGCACCA-3′) and S-HMC-(D1 + D2A)-R (5′-GGCTGCAGTGCACGTTGGGACTGTATA-3′), and S-HMC D2B + D3 primers S-HMC-(D2B + D3)-F (5′-CGGAGCT CACGTGCAGTACTATGGAG-3′) and S-HMC-(D2B + D3)-R (5′-GGCTGCAGGAAGAGTTGTAAGCTTGAATC-3′), were designed based on the nucleotide sequence of S-HMC cDNA from Genbank database (X82502.1) for the amplification of S-HMC and its fragments including S-HMC D3 (1752–2528, 777 bp), S-HMC D1 + D2A (501–1572, 1,072 bp), and S-HMC D2B + D3 (1566–2528, 963 bp), respectively. SacI and PstI restriction sites were added to the 5′ and 3′ ends of the primers. Each PCR amplification reaction consisted of 0.2 µg of cDNA, 0.4 μM of primers, 0.2 mM dNTP, 3.0 mM MgCl_2_, and 1 U of Pfu DNA polymerase in 25 µL of the reaction mix, as recommended by the manufacturer. Two combined amplification cycles with conditions 60 s 94°C, 60 s 52°C, 60 s 72°C (5 cycles), 60 s 94°C, 60 s 58°C, 60 s 72°C (33 cycles) were carried out using an UNO-Thermoblock (Biometra, Göttingen, Germany). The PCR products were extracted and inserted into pQE-32 vector (QIAGEN) and then transformed in *E. coli* XL1-Blue MRF cells. Positive clones were picked and confirmed by sequencing. Expression of S-HMC and its fragments were treated with 1 mM IPTG and grown for 4 h. The recombinant proteins were purified by nickel-based resins, and then used to investigate their reactivity with anti-human Ig and agglutinating activity with bacteria or erythrocytes.

### Gene Cloning, Expression and Protein Purification of *E. coli* K12’s OM Proteins

Bacterial clones pET-32a-OmpC, -FadL, -OmpW, -OmpT, -OmpA, -OmpX, -LamB, and -Dps were obtained from collections in our laboratory and their corresponding antibodies were ordered from Wenta Bio Sci Tech Corp (Ji’an, China). The specificity of these antibodies was validated using the gene-deleted strains of the proteins in our previous publication (36). The recombinants were expressed with IPTG inducement and then identified by 1-DE. The resulting *E. coli* K12 harboring pET-32a-OmpC, -FadL, -OmpW, -OmpT, -OmpA, -OmpX, -LamB, and -Dps were cultured in 100 mL LB media and induced by IPTG. The recombinant proteins (His_6_-Omps) were purified by affinity chromatography using Ni-NTA resin (Qiagen) and then used to investigate their reactivity with *L. vannamei* HMC.

### Preparation of HMC-M or HMC-T Antisera and Purification of the Different HMC Components Using Affinity Chromatography

First, the purification of HMC-M, HMC-G, or HMC-A was by affinity chromatography as previously described (26). Goat anti-human IgM, IgG, or IgA (5 mg each) antiserum was purified by the methods of ammonium sulfate precipitation and ion-exchange chromatography. The purified antibody was then covalently coupled to CNBr-activated sepharose 4B (0.5 mL) using carbonate buffer (0.5 M NaCl, 0.1 M NaHCO_3_, pH 8.3) and the matrix packed into a 1.0 mL syringe. Pooled shrimp sera was loaded onto the affinity column and incubated at 4°C for 24 h. After washing with 15 column volumes of PBS (0.05 M pH 7.0), the bound proteins were eluted with 3 mL of glycine-HCl buffer (0.1 M pH 2.2) and the eluates were neutralized with 300 µL of Tris-HCl buffer (1 M pH 8.0). The eluted proteins (HMC-M, HMC-G, or HMC-A) were concentrated by PEG 20,000, separated by 1-DE, and then transferred onto nitrocellulose membranes for Western blot and agglutination activity analysis. Secondly, the HMC-M or HMC-T antisera were prepared as previously described (26). Any band retained within the affinity chromatography with goat anti-human IgM and recognized by the same antibody in Western blot analysis, was stained, cut and used to prepare HMC-M by elution. Protein concentrations were determined according to Bradford’s method (37). The purified HMC-M was used as an antigen to immunize New Zealand white rabbits by multiple subcutaneous injections. Anti-HMC-M polyclonal antibodies were harvested from the rabbits’ serum when the antibodies titer was high. Meanwhile, shrimp HMC purified from *L. vannamei* sera by the method of molecular sieve chromatography (38), was used to prepare the HMC-T antisera under the same conditions. Thirdly, HMC-T was purified by affinity chromatography as described above. Next, different HMC components bound with OmpA, OmpC, OmpT, OmpX, or DpS were purified from HMC-T using the above affinity chromatography strategy, in which, recombinant Omps of *E. coli* K12 including OmpA, OmpC, OmpT, OmpX, or DpS were used as affinity ligands, respectively. Finally, concentrations of all the HMC fractions were determinated by the Bradford method (37), filtered (0.22 µm) and then used for immune activity analysis.

### One-Dimensional Sodium Sulfate-Polyacrylamide Gel Electrophoresis and Western Blot Analysis

The immune proteins which bound with the bacteria *V. parahaemolyticus, V. fluvialis, P. fluorescens*, and *E. coli* K12 in shrimp sera, as well as shrimp sera, purified recombinant S-HMC and its fragments, affinity chromatograph purified HMC fractions (80 µg/mL), i.e., HMC-T, HMC-M, HMC-A, and HMC-G were separated on 1-DE using a 3% stacking gel (pH 6.8) and a 10% separating gel (pH 8.9) in Tris-glycine buffer (pH 8.3) and then transferred onto PVDF membranes for 6 h at 200 mA in transfer buffer (25 mM Tris, 0.1 M glycine and 20% methanol). Membranes were blocked for 60 min with 5% skimmed milk in TBS (20 mM Tris, 150 mM NaCl, pH 7.4) at 37°C, then incubated first with primary antibodies (rabbit anti-HMC-M or -HMC-T, goat anti-human IgM, IgG or IgA, rabbit anti-*E. coli* OmpC, FadL, OmpW, OmpT, OmpA OmpX, LamB, or Dps) for 2 h at 28°C, followed by second antibodies (goat anti-rabbit IgG-HRP or rabbit anti-goat IgG-HRP at a dilution of 1:1,000 for 1 h) at 37°C in TBST (20 mM Tris, 150 mM NaCl, 0.5% Tween-20, pH 7.4) containing 5% skimmed milk on a shaker. The membranes were developed with 3′3-diminobenzidine (DAB) substrate.

### Two-Dimensional Polyacrylamide Gel Electrophoresis, 2D Western Blot, and Far-Western Blot Analysis

Two-dimensional polyacrylamide gel electrophoresis (2-DE) analysis of the outer member proteins of *E. coli* K12 and the different HMC fractions (HMC-T and HMC-M) was performed as previously described (39). In brief, a total of 40 µg of bacterial or HMC proteins in rehydration buffer (containing 7 M urea, 2 M thiourea, 4% CHAPS, 0.2% DTT, and 3.4 mL of IPG buffer, pH 3–10) was used to rehydrate the IPG strip (7 cm, pH 3–10 for bacterial proteins and pH 4.7–5.9 for HMC fractions; Bio-Rad, Hercules, CA, USA) for 16 h. The isoelectric focusing was performed at a constant temperature of 20°C using a continuous increase in voltage (up to 4,000 V) until 32,000 V. Prior to the second dimension, the focused IPG was incubated for 15 min in an equilibration buffer containing 20% w/v glycerol, 2% SDS, 0.375 M Tris-HCl (pH 8.8), 2% DTT, and then further equilibrated for 15 min in a similar buffer in which 2% DTT was replaced with 2.5% of iodoacetamide. The strip was placed on top of a 10% 1-DE gel. Low-melting point agarose was used to cover the IPG strip and a filter paper. The following procedures including 1-DE separation of proteins and Western blot assay were carried out under the same conditions as described for 1-DE and Western blot above. For Far-Western blot analysis, *E. coli* K12 OM proteins were separated by 2-DE and transferred onto nitrocellulose membranes. The membranes were incubated with HMC and anti-HMC-T as a bridge protein and the primary antibody, respectively, and developed with DAB substrate as described above.

### Image Analysis

The 2-DE gel images were analyzed with the PDQuest software version 8.0 (Bio-Rad, Hercules, CA, USA). Comparative analysis of protein spots was done by matching corresponding spots across different gels. Each of the matched protein spots was rechecked manually. After normalization with total intensity of all spots present in each gel, the intensity of individual spots was subjected to statistical analysis, comparing HMC-M with HMC-T. Proteins were considered to be differentially expressed between the two groups based on the following criteria (1): *p* < 0.05 (2), significantly increased or decreased when mean value is more than 2.0-fold (3), consistent change in all replicates.

### Mass Spectrometry

The protein bands (bacteria-binding proteins), or spots including bacterial proteins bound to HMC, and HMC-T and HMC-M, were excised from the 1-DE or 2-DE gels and digested with trypsin as previously described (39). Briefly, the bands or spots were washed several times with 50% acetonitrile, followed by drying the gels in a vacuum freeze dryer. The cysteine reduction and alkylation steps consisted of incubation first in 10 mM DTT for 30 min, 55 mM iodoacetamide for 45 min in the dark at room temperature. The gels were then dried again and rehydrated in a minimal volume of 50 mM NH_4_HCO_3_ pH 8.0 containing 5 mM CaCl_2_ and 12.5 ng/µL sequencing grade modified trypsin for 45 min on ice. The excess liquid was removed and the pieces of gel were immersed overnight in the same buffer (without enzyme) at 37°C. The gels were extracted in 20 µL of 20 mM NH_4_HCO_3_ pH 8.0 followed by three extractions in 20 µL 5% formic acid in 50% acetonitrile. The resulting pooled eluates were dried prior to analysis by mass spectrometry. 0.5 µL of the peptide mixture was mixed with the matrix a-cyano-4-hydroxycinnamic acid (1:1) and spotted onto a stainless steel MALDI plate. Mass spectra were obtained using the ABI 4700 Proteomics Analyzer MALDI-TOF-TOF mass spectrometer (Applied Biosystems, Foster City, CA) operating in a result-dependent acquisition mode. Peptide mass maps were acquired in reflectron mode (1-keV accelerating voltage) with 1,000 laser shots per spectrum. Six external standards (mass standard kit for the 4700 Proteomics Analyzer calibration mixture, Part Number 4333604; Applied Biosystems, Foster City, CA, USA) were used to calibrate each spectrum to a mass accuracy within 50 ppm. Selected peptide masses were submitted to Mascot (http://www.matrixscience.com/cgi/search_form.pl?FORMVER=2&SEARCH=PMF) for NCBInr databases search.

### Far-Dot-ELISA

Far-Dot-ELISA analysis of HMC-T and HMC-M was performed according to routine procedure. In brief, nitrocellulose (NC) membrane was cut into desired size, soaked in TBS (20 mM Tris, 150 mM NaCl, pH 7.4) for 5 min and then allowed to dry on a filter paper. 1 µL aliquots of each HMC fractions (0.5 mg/mL) was spotted onto the NC membrane using a pipet. After drying, the NC membrane was blocked at 37°C for 1 h with 5% skimmed milk in TBS, rinsed 3× with TBS for 5 min, and then separately reacted with six *E. coli* K12 Omps, i.e., OmpT, OmpW, OmpX, OmpC, OmpA, and FadL (1 mg/mL) for 1 h at room temperature. After rinsing three times with TBS for 5 min each time, the membrane was reacted with a 1:200 dilution of rabbit anti-OmpT, -OmpW, -OmpX, -OmpC, -OmpA, or -FadL antisera for 2 h at room temperature, respectively, and then incubated with secondary antibodies (goat anti-rabbit IgG-HRP at a dilution of 1:1,000 for 1 h) at 37°C in TBST containing 5% skimmed milk on a gently rocking shaker. The membrane was washed as above and developed with DAB substrate. The precipitated dots were scanned and analyzed by the analysis system of GDS8000PC.

### Agglutination and Hemagglutination Activity Assays

*V. parahaemolyticus, V. fluvialis, E. coli* K12, or chicken erythrocytes were used for agglutination or hemagglutination analysis as previously described (26). The bacteria were cultured overnight at 28 or 37°C, harvested, washed and diluted to 10^8^ CFU/mL in TBS-Ca^2+^ (0.05 M Tris, 0.75% NaCl, 0.05 M CaCl_2_, pH 7.4). Chicken erythrocytes were washed three times with TBS (50 mM Tris, 150 mM NaCl, pH 7.4), centrifuged at 500*g* for 5 min and then diluted to 0.5% erythrocytes suspension in TBS-Ca^2+^. The agglutination or hemagglutination activities of HMC fractions bound to bacteria, HMC fractions purified by affinity chromatography covalently coupled with goat anti-human IgM/IgG/IgA or rabbit anti-shrimp HMC-T/HMC-M or recombinant Omps of *E. coli* K12, and recombinant HMC and its fragments were measured by mixing 20 µL bacteria or chicken erythrocytes with 20 µL of twofold serial dilutions of each HMC fractions at 37°C for 30 min. Agglutination was viewed by light microscopy and the agglutination or hemagglutination titer was defined as the reciprocal of the highest dilution of the samples tested.

### Antibacterial Assays

Shrimp sera from *L. vannamei* infected with *E. coli* K12 BW25113 and its genetically modified strains with gene deletion and HMC fractions (HMC-M and HMC-T) obtained from affinity chromatography were filtered (0.22 µm) and then used for antibacterial assay. First, the antibacterial activity was determined by the number of bacterial colonies growing or present on the Petri dish. Bacteria including *V. alginolyticus, V. parahaemolyticus, V. harveyi, V. fluvialis, V. anguillarum, A. hydrophila, A. sobria, S. aureus, E. coli* K12 BW25113, and its genetically modified strains with gene deletion of OmpX, OmpC, OmpT, OmpA, OmpW, FadL, and Tsx were grown in LB or Broth medium overnight, collected, washed with 0.85%NaCl three times, and then resuspended and diluted with TBS-Ca^2+^ buffer to 600–6,000 CFU/mL. Meanwhile, the eluted HMC fractions were diluted 5 folds with 50 mM Tris-HCl pH 7.2 to make the salt concentration suitable for the normal growth of bacteria before they were serially diluted with TBS buffer (20 mM Tris, 150 mM NaCl, pH 7.4). 100 µL each of the serially diluted HMC fractions was mixed with half volume of the corresponding bacteria suspension. After the mixture was incubated at room temperature for 30 min, 50 µL of incomplete nutrient medium (0.04% bactotryptone, 0.02% yeast extract, 0.75%NaCl, 50 mM CaCl_2_, 50 mM Tris-HCl, pH 7.2) was added and incubated for another 1.5 h. Bacteria numbers were measured by counting the colony-forming units after pre-incubated mixture of bacteria suspension and protein samples was spread on LB Petri dish and then incubated for 12 h at 37°C. A mixture of the diluted elution buffer and bacteria was cultured as control. Secondly, 50 µL of bacterial suspension (10^7^–10^8^ CFU/mL) was spread on LB Petri dish, and then 50 µL of HMC-M or HMC-T (200 µg/mL) was added in an Oxford cup. After incubating for 12 h at 37°C, the diameter of inhibition zone was measured. Each treatment was repeated three times.

### Hemolytic Activity Assays

Hemolytic activities of HMC-T and HMC-M, as well as oxidized HMC-T and HMC-M, were determined by measuring the absorbance at 540 nm as previously described (27, 40). Briefly, 125 µg/mL of different HMC fractions (0.9 mL) were mixed with 0.5% (v/v) of four types of human erythrocytes suspension (0.3 mL). After incubation at 37°C for 1 h, undamaged cells and cell debris were removed from the samples by centrifugation at 3,500*g* for 10 min, and hemolytic activity was determined by measuring the absorbance of supernatants at 540 nm. The supernatant from the 0.5% (v/v) erythrocytes suspension treated with double distillated water was used as a 100% hemolysis control, while that treated with PBS-Ca^2+^ (0.01 M, pH 6.0) was used as a 0% hemolysis blank. All samples were prepared in triplicate. To further compare the hemolytic activity between HMC-T and MHC-M, kinetic analysis of human erythrocytes from blood group A, B and O types was performed. 0.5% (v/v) human erythrocyte suspension (10 µL) was mixed with HMC fractions (10 µL, 200 µg/mL for groups A and O, or 125 µg/mL for group B) on a slide. After incubation at 37°C for 15, 30, 45, and 60 min, digital photomicrographs were taken with an Olympus BH-2 microscope (Olympus Company, Tokyo, Japan).

### Determination of Phenoloxidase Activity

0.5 mg/mL of HMC fractions (100 µL) was mixed with 0.01 M trypsin (100 µL). After incubation at 37°C for 30 min, 0.01 M dopamine (100 µL) and 0.1 M potassium phosphate buffer (pH 6.0, 3 mL) were added before detecting the absorbance at 490 nm. Double distillated water and 5 mg/mL shrimp sera were used as negative and positive controls, respectively. Phenoloxidase activity was estimated as the increment in the rate of absorbance at 490 nm with an increase of 0.001 per minute taken to be 1 U. All samples were prepared in triplicate.

### Total Glycan Measurement

The carbohydrate content of HMC fractions was determined using the colorimetric method previously described (41) with modification. Briefly, 0, 100, 200, 300, and 400 µL of 0.01 M standard glucose solutions were added to tubes, and made up to a final volume of 500 µL using double distillated water. 300 µL of 6% (m/v) phenol and 1.5 mL sulfuric acid were added immediately. After incubation at room temperature for 25 min, the absorbance at 490 nm was determined. A standard curve was constructed using sugar concentration as ordinate and the absorbance as abscissa. Using 200 µL of 0.25 mg/mL HMC fractions, the total glycan content of the HMC fractions was extrapolated from the standard curve using their absorbance values.

### Carbohydrate Detection

Tricine-SDS-PAGE, high-performance liquid chromatography (HPLC), lectin blot, and Far-Dot-ELISA assays were used to compare the difference in glycosylation between HMC-T and HMC-M. First, Tricine-SDS-PAGE analysis of HMC fractions treated with trypsin was used as previously described with modification (42). Briefly, 4 mg of HMC-T or HMC-M was treated with trypsin (trypsin:HMC ratio 1:50) for 3 h at room temperature in 0.1 M pH 9.0 NH_4_HCO_3_ buffer. After dialysis with 0.01 M pH 7.4 PBS, the digested peptides from the HMC fractions were separated on Tricine-SDS-PAGE using a 4% stacking, 10% spacing, and 16.5% resolving gel. Secondly, the peptides were further analyzed using HPLC. Separation was achieved using a C18 reversed-phase chromatography column (25 cm × 4.6 cm) with a linear gradient of 90 to 10% ACN (containing 10–90% of 0.1% TFA), which encompassed 60 min. The eluant was monitored by UV detection at 214 nm. Next, lectin blot was performed as described by Figueroa-Soto et al. (43) with modification. In brief, 10 µg of HMC-T and HMC-M were separated by 1-DE and then transferred onto a PVDF membrane as described above. The membrane was blocked with 2% PVP360 (polyvinylpyrrolidone 360) in 0.05 M TBS (0.5 M NaCl, pH 7.5) for 2.5 h at 25°C, then incubated with four biotinylated lectins [concanavalin A (CON-A), dolichos biflorus agglutinin (DBA), peanut agglutinin (PNA), or ulex europaeus agglutinin (UEA)] (1:5) in 0.02 M TBS (0.5 M NaCl, pH 7.5) for 1 h at 37°C, followed by peroxidase-conjugated avidin (1:1,000) in the same buffer at 37°C for 40 min. Carbohydrates were detected by incubating the membrane with a substrate (DAB). The precipitation dots were scanned and analyzed by the GDS8000pc gel image formation analysis system. Finally, the characterization of HMC fractions binding to the lectin of CON-A was further investigated by Far-Dot-ELISA. 1 µL aliquots of CON-A (1:1 or 1:10) was spotted onto the prepared nitrocellulose membrane. After being blocked at room temperature for 2 h with 2% PVP 360 in TBS, the membrane was treated with 0.1 mg/mL of HMC-T or HMC-M for 2 h at room temperature, and then incubated with rabbit anti-shrimp HMC-T antisera (1:1,500) and goat anti-rabbit IgG-HRP (1:3,000) antibodies at room temperature for 1 h, respectively. The rest of the procedure is same as outlined above in the section of HMC fractions binding to *E. coli* K12 Omps.

### Glycan Oxidation Test

The carbohydrate epitope of the HMC fractions was destroyed by periodate oxidation according to Pagano et al. (44). 125 µg/mL of HMC-M or HMC-T was oxidized with 15 mM KIO_4_ in sodium acetate buffer, pH 4.5, at room temperature for 30 min in the dark, followed by dialysis with 0.01 M PBS at pH 7.4 overnight. Both the oxidized HMC fractions and non-treated HMC fractions were further analyzed using the hemolytic activity assay as described above.

### Statistical Analyses

All the statistical analyses were performed using SPSS version 22.0 (SPSS Inc., Chicago, IL, USA) and GraphPad Prism Version 5 software. Data were presented as mean ± SD/SE. Statistical differences were estimated by one-way ANOVA and data were considered statistically significant at *p* < 0.05.

## Results

### Hemocyanin Is the Major Bacteria-Binding Protein in the Hemolymph of Shrimp

To uncover the frontline recognition and defense molecules in shrimp, four inactivated bacterial strains (*V. parahaemolyticus, V. fluvialis, P. fluorescens*, and *E. coli* K12) were used as baits to pull-down proteins in freshly isolated hemolymph of shrimp. Two dominant bands (migrating around 75 and 77 kDa) were found in all the four bacterial strains, while no such bands were detected in bacteria incubated with PBS (Figure S1A in Supplementary Material). The two protein bands were identified as HMC by mass spectrometry (Figure S1B in Supplementary Material), which was further validated by Western blot analysis using rabbit anti-HMC antibodies (Figure S1C in Supplementary Material).

We have previously shown that HMC displayed immunogenic ability as it bound to pathogenic bacteria inducing agglutination (26). Therefore, the two bands were recovered from the gels and incubated with *E. coli* K12 and *V. fluvialis* (Figure S1D in Supplementary Material), both of which showed clear agglutination activity of 0.54 and 0.27 µg/mL, respectively. These results indicate that HMC serves as an innate immune defense protein of *L. vannamei* in response to bacterial pathogens.

### Convergent Evolution between Domains of HMC Confers C-Terminal as an Ig-Like Domain

**Figure 1:**
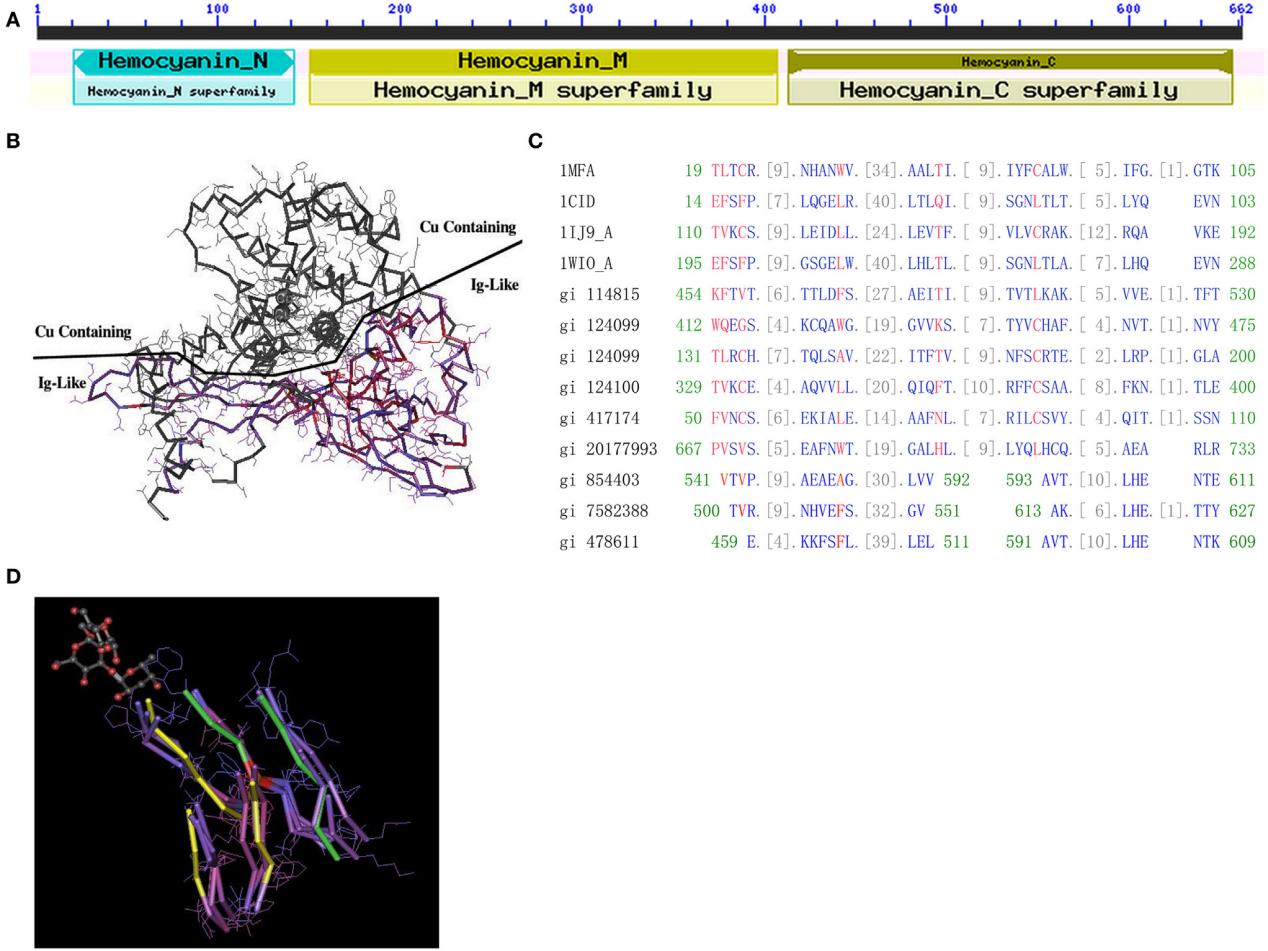
**Protein structure of hemocyanin (HMC)**. **(A)** The conserved domains of *L. vannamei* S-HMC (X82502.1/gi|854403). The domain is divided into D1 (HMC N, length 20–148, all-alpha conserved domain), D2 (HMC M, length 150–407, copper containing domain), and D3 (length 413–657, Ig-like conserved domain). **(B)** The three-dimensional structure of S-HMC, indicating that D2 and D3 are exposed on each side of the S-HMC structure, with both having an equal chance of exposure to the environment. **(C)** Conserved domain alignment of the Ig domain (cd00096) with HMC D3 of gi|854403, gi|7582388, and gi|478611 from shrimp. **(D)** A tertiary structure of an Ig skeletal domain, showing a skeleton structure with five beta-folding units which are made up of several overlapping Ig domains (NCBI Conserved Domains id: Cd00096). Out of the five folding units, amino acid residues 541–592 and 593–611 of S-HMC D3, are matched on three of the five beta-folding units (marked yellow) and on the other two (marked green).

Our research group and others have shown that HMC is the major bacteria-binding protein in the hemolymph of shrimp, which induces bacterial aggulutination and hemolysis (26, 27). However, the mechanisms by which HMC recognizes bacterial invaders and initiates an immune response were largely unknown. The immunogenic ability of HMC appears to be synanomous to human immunoglobulins (hIg), such as recognition of bacterial pathogens (26). Unfortunately, we could not find the homology between HMC and human hIg, which suggest that HMC may not have fully evolved into hIg. *L. vannamei* HMC is a large protein complex (hexamer or dodecamer), composed of two subunits, i.e., a large subunit (77 kDa, L-HMC) and a small subunit (75 kDa, S-HMC). Both subunits contain three domains: D1 (all-alpha domain, residue: 20–148), D2 (copper containing domain, residue: 150–407), and D3 (Ig-like domain, residue: 413–657) (Figure 1A). We thought that local alignment may help us to resolve the issue of homology, so we obtained the 3D structure of HMC (PDB id: 1HC1) and showed equal chance to environmental stresses in space between D3 and D2 domains since they were exposed on each side of the HMC structure (Figure 1B). To examine the genetic distances among the three domains of HMC at different levels of evolution, protein sequences of S-HMCs from six species: *L. vannamei* (AccNum X82502), *P. vulgaris* (AccNum AJ344361), *P. leniusculus* (AccNum AJ272095), *H. americanus* (AccNum AY193781), *P. elephas* (AccNum AJ516004), and *G. roeseli* (AccNum AJ937836), were extracted from databases and used for domain alignments. Interestingly, amino acids variations between D3 and D1 or D2 were significant among all the examined species (both *p* = 0.000) but not for D1 and D2 with mean values of 0.447528 for D1, 0.226219 for D2, and 0.666782 for D3 in JTT distance. Furthermore, Mann-Whitney U test also showed significant difference between D3 and D1 (*p* = 0.001), and D3 and D2 (*p* = 0.000) (Table S1A in Supplementary Material). In addition, the results from accumulation of non-synonymous (Ka) substitutions analysis showed a similar result, as the Ka value of D3 (0.35462) was much higher than that of D1 (0.25106, *p* = 0.001) and D2 (0.12775, *p* = 0.000) (Table S1B in Supplementary Material). Taken together, these results suggest that the D3 domain might have evolved faster than the other two domains under selection pressure thereby showing more genetic variation and diversity.

The likelihood of genetic variation between HMC D3 and hIg suggests some evolutionary relationship between these two molecules. Therefore, we aligned the D3 domain with three HMC-related domains (Pfam00129, Pfam00929, and Pfam00993) and four Ig-related domains (Cd00096, Cd00098, Cd00099, and Cd00931). The alignment of D3 to these domains showed 9 to 15 consecutive homologous units (amino acids) (Figure 1C). The best-matched sequence was Cd00096, which is from Ig, T-cell receptor (TCR), and cluster of differentiation molecules (CD) and constituted 34 units. Among the 34 units, 26 units of *L. vannamei* HMC D3 were matched (76.5%), with the longest matching being 15 units (44.1%). Similar alignment results were obtained with D3 of L-HMC (gi|7582388 and gi|478611) (Figure 1C).

Next, multiple protein sequence alignment of D3 (S-HMC) to Ig superfamily molecules from *Homo sapiens, Drosophila melanogaster*, and *Caenorhabditis elegans* showed highly homologous amino acid sequences. D3 shared three conserved regions with the variable regions of IgM heavy chain (gi|4995350), VH3 family of IgM heavy chain (gi|33318922), and IgA heavy chain (gi|7161007). Their identical frequencies were 24% (8/33), 43% (10/23), and 54% (7/13), respectively (Figure S2A in Supplementary Material). In addition, it shared a conserved region with 49 amino acid residues with the variable region of hIg κ chain (gi|722524), of which 38.8% (19/49) residues were identical (Figure S2B in Supplementary Material). High similarity was also found in the segments between the L-HMC of *L. vanammei* (AJ250830.1/gi|7414468) and several hIg variable regions of the Ig λ light chain (gi|3143574), and Ig κ chain (gi|722486) (Figure S2C and Table S1C in Supplementary Material). Therefore, several regions of HMC share local similarity with the hIg variable regions.

Finally, the relationship between D3 and Ig domain patterns from 3D structures was examined. Figure 1D shows an Ig skeleton structure with five beta-folding units, which are made up of several overlapping Ig domains (NCBI Conserved Domains id: Cd00096). Among the five folding units, three strands (marked yellow) and two strands (marked green) are matched with the amino acid residues 541–592 and 593–611 of D3 (gi|854403), respectively (Figure 1D). These data suggest that the D3 domain shares a similar domain with Ig-related molecules in terms of sequence and 3D structure.

### HMC D3 Domain Is Highly Variable

**Figure 2:**
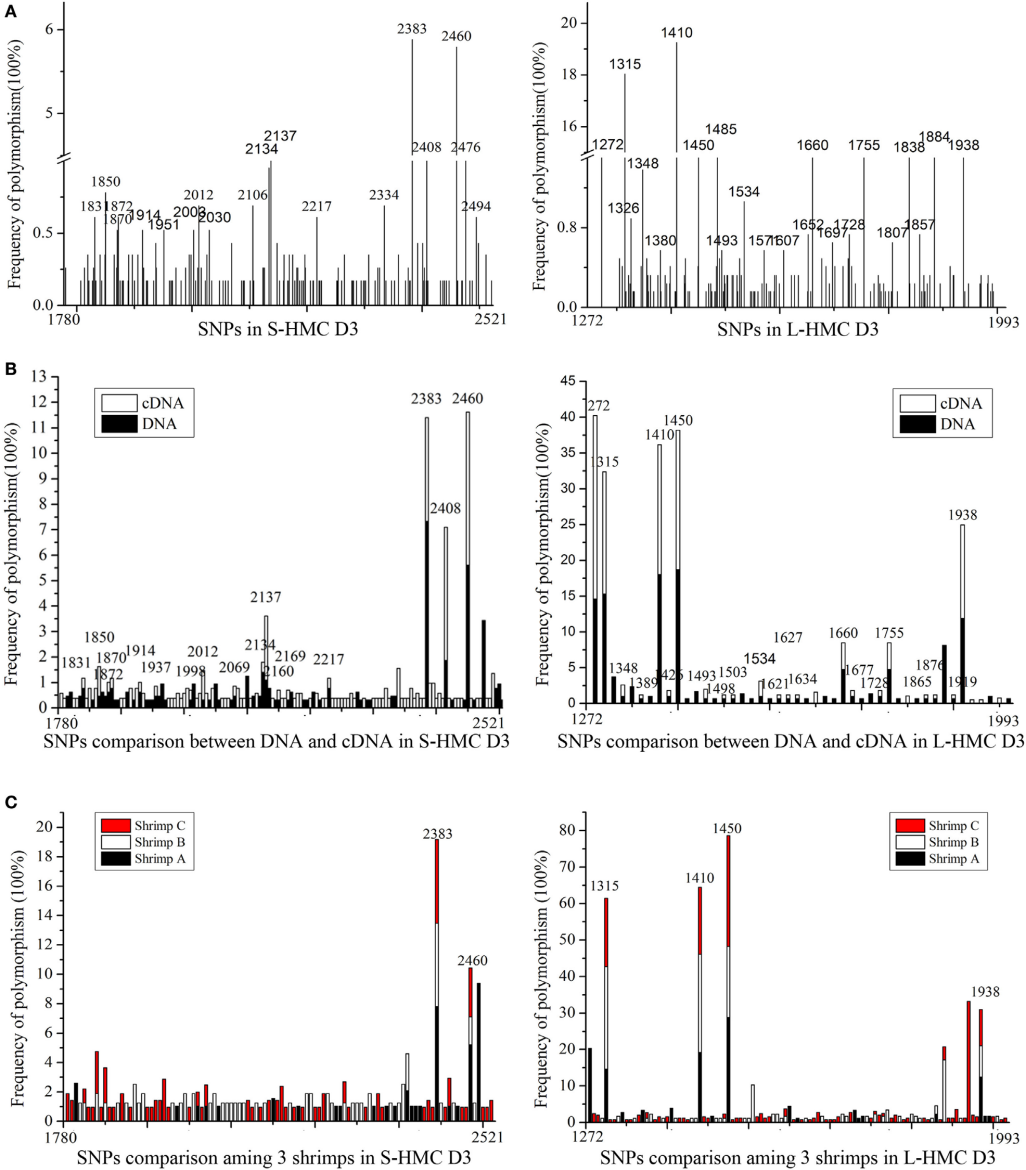

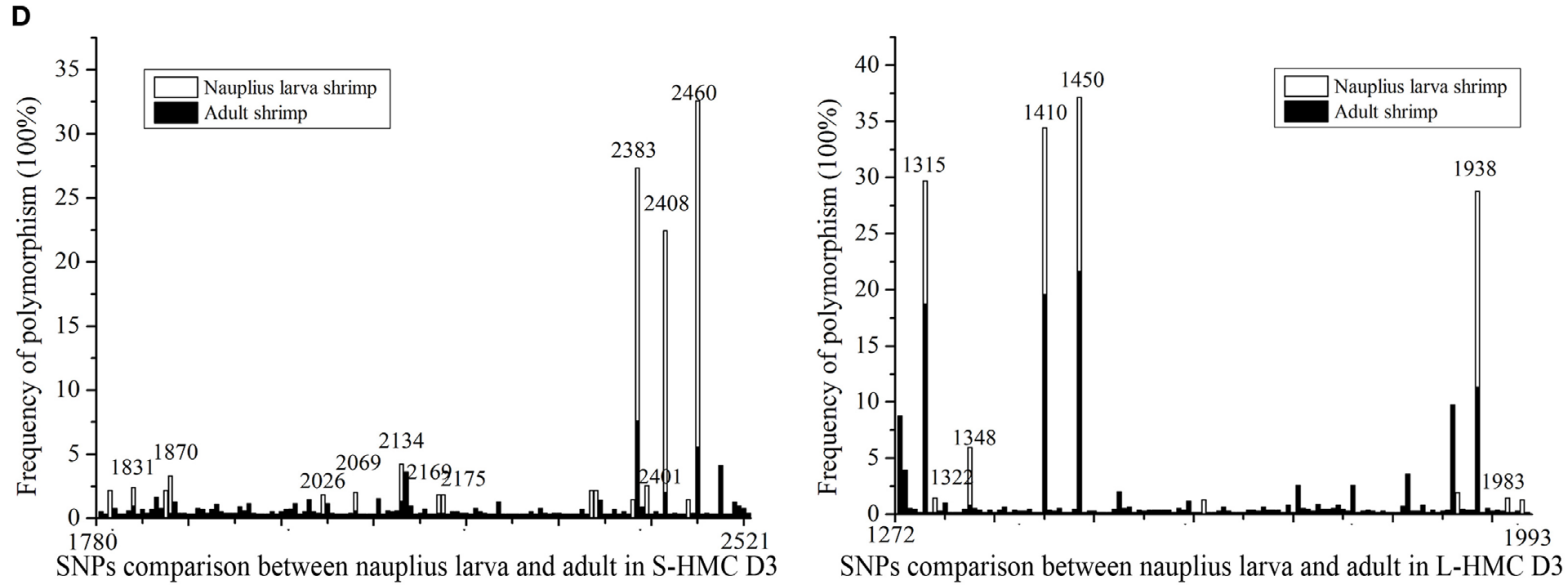
**SNPs of hemocyanin (HMC) D3 domain from *L. vanammei***. **(A)** SNPs analysis of three shrimps and nauplius larvae at two levels (cDNA and genomic DNA). **(B–D)** Comparison of SNPs between genomic DNA and cDNA, three shrimps’ heaptopancreas, nauplius larva and three adult shrimps, respectively, based on the data of **(A)**.

We reasoned that the HMC D3 domain might show the same diversity of genes as the human Ig variable region, due to somatic point mutations (45–48). To evaluate this, we sequenced genes encoding the D3 domain of S-HMC and L-HMC from four organs, i.e., heart, stomach, gill, and heaptopancreas, in a single shrimp (designated shrimp A). Similarly, sequencing of the D3 domain of S-HMC and L-HMC from heaptopancreas of the other two shrimps (designated shrimp B and C) and that of nauplius larva was carried out so as to better understand the mutant phenotypes. Twenty-eight genomic DNA and cDNA plasmid libraries were constructed (i.e., 16 from the four organs of shrimp A, 8 from heaptopancreas of shrimps B and C, and 4 from nauplius larva). In investigating the mutant phenotypes, 1,157 clones of S-HMC and 1,231 clones of L-HMC obtained from the 28 libraries of 3 shrimps and nauplius larva were sequenced. Upon analysis of the sequencing data, 141 SNP sites (61 sites in genomic DNA and 97 sites in cDNA) and 126 SNP sites (88 sites in genomic DNA and 71 sites in cDNA) were identified in S-HMC D3 and L-HMC D3, respectively (Figure 2A; Figure S3 in Supplementary Material). Out of these, only 17 S-HMC SNPs (1831, 1850, 1870, 1872, 1914, 1937, 1998, 2012, 2069, 2134, 2137, 2160, 2169, 2217, 2238, 2408, and 2460) and 33 L-HMC SNPs (1272, 1315, 1322, 1348, 1389, 1410, 1426, 1449, 1450, 1493, 1498, 1503, 1504, 1526, 1534, 1571, 1621, 1627, 1634, 1652, 1660, 1677, 1717, 1728, 1755, 1807, 1865, 1876, 1884, 1919, 1938, 1947, and 1983) were identical between the genomic DNA and cDNA (Figure 2B; Figure S3 in Supplementary Material), indicating that there was variation between the S-HMC D3 SNPs (124/141) and L-MHC D3 SNPs (93/126). Similarly, a comparison of the SNPs among the heaptopancreas of shrimp A, B and C indicated that only two SNP sites (2383 and 2460) and four SNP sites (1315, 1410, 1450, and 1938) were identical in the S-HMC D3 and L-HMC D3 (Figure 2C; Figure S3A–D in Supplementary Material). Also, about 64% (1831, 1870, 2026, 2069, 2134, 2169, 2175, 2383, 2401, 2408, and 2460) and 70% (1315, 1322, 1348, 1410, 1450, 1938, and 1983) of S-HMC D3 and L-HMC D3 SNPs in nauplius larva were identical to those of SNPs observed in the adult shrimps (Figure 2D; Figure S3 in Supplementary Material). Noticeably, SNP frequency analysis at genomic and cDNA levels revealed that most were low from 0.17 to 3.37%, while only two SNP sites (2383 and 2460) of S-HMC D3 and 7 SNP sites (1272, 1315, 1348, 1410, 1450, 1884, and 1938) of L-HMC D3 were a bit higher, i.e., 5.79–19.25% (Figure 2; Figure S3 in Supplementary Material). In addition, 10 β-actin clones were included as negative control to exclude potential PCR and sequencing errors. Taken together, these results imply that the HMC D3 possesses extensive SNPs, which is tissue, individual and developmental stage independent, and has a similar pattern to that of human Ig variable regions.

### HMC D3 Binds Bacteria and Reacts with Anti-Human Ig

**Figure 3:**
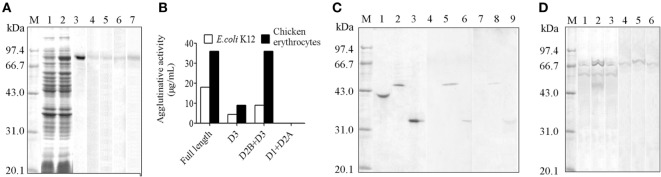
**Diversity of hemocyanin (HMC) structure**. **(A)** One-dimensional sodium sulfate-polyacrylamide gel electrophoresis (1-DE) and Western blot analysis of recombinant S-HMC expression. M, Protein marker; 1–3, 1-DE of IPTG-uninduced (1) and IPTG-induced (2) S-HMC-XL1-Blue MRF, and purified recombinant S-HMC (3); 4–7, Western blot of recombinant S-HMC recognized by anti-human immunoglobulin (Ig) G (4), IgA (5), or IgM (6) and anti-shrimp HMC-T (7). **(B)** Agglutinative or hemagglutinative activity (μg/mL) of recombinant full-length S-HMC and its three fragments reacted with *E. coli* K12 and chicken erythrocytes. **(C)** Western blot analysis of recombinant S-HMC fragments, D1 + D2A, D2B + D3, and D3. M, Protein marker; 1–3, 1-DE of purified recombinant D1 + D2A, D2B + D3, and D3, respectively; 4–9, Western blot of purified recombinant D1 + D2A, D2B + D3, and D3 using anti-shrimp HMC-T (4–6), and anti-human IgM (7–9). **(D)** Western blot analysis of HMC as an antigen recognized by anti-human IgM, IgG, or IgA using affinity chromatograph. M, Protein marker; 1–3, 1-DE for samples from affinity chromatograph by anti-human IgM, IgG, or IgA, respectively; 4–6: Western blot for samples (1–3) recognized by anti-human IgM, IgG, and IgA, respectively.

The demonstration of HMC D3 diversity led us to reason that probably HMC D3 plays a key role in immune recognition and reactivity. To examine this, recombinant S-HMC and its three recombinant fragments, D1 + D2A, D2B + D3, and D3 were generated. The recombinant S-HMC was recognized by anti-human IgG, IgA, IgM, or anti-HMC-T (Figure 3A). When they were separately mixed with *E. coli*, recombinant D1 + D2A showed no agglutinative activity with the bacteria, whereas distinguishable agglutinative activities were obtained with recombinant S-HMC, D2B + D3, or D3 fragments, i.e., 18, 9, and 4.5 µg/mL, respectively (Figure 3B). Similar results were obtained when the same recombinant proteins were reacted with chicken erythrocytes (Figure 3B). These results indicate that HMC D3 is essential for binding to bacteria as well as initiates agglutination reaction with bacteria and erythrocytes.

Meanwhile, when the three recombinant domain fragments were purified as antigens and used for Western blot analysis, it was only the purified recombinant D3 and D2B + D3 fragments that showed up with goat anti-human IgM, but not D1 + D2A (Figure 3C). These findings further suggest that HMC D3 and human Ig have overlapping or similar epitopes.

### Functional HMC Contains at Least Two Subpopulations

Genetic variability of the D3 domain may have resulted in heterogeneous population of HMC, which lead to functional diversities of HMC. We previously showed that a subpopulation of HMC is recognized by anti-human IgG or IgA (26, 49), but whether this phenotype is related to the functional diversity of HMC is unknown. We therefore isolated subpopulations of HMC based on their ability to interact with different Ig molecules. Freshly isolated HMC was subjected to affinity-chromatograph using anti-human IgM, IgG, or IgA, and the eluted proteins were used for Western blot validation. Only one band mainly at 75 kDa (S-HMC) or 77 kDa (L-HMC) was readily recognized by anti-human IgM, IgA, or IgG (Figure 3D). Actually, all of the bands were identified as HMC by mass spectrometry (data not shown).

### HMC Subpopulation (HMC-M) Recognized by Anti-IgM Is the Major Component for Aggulutination and Hemolysis

**Figure 4:**
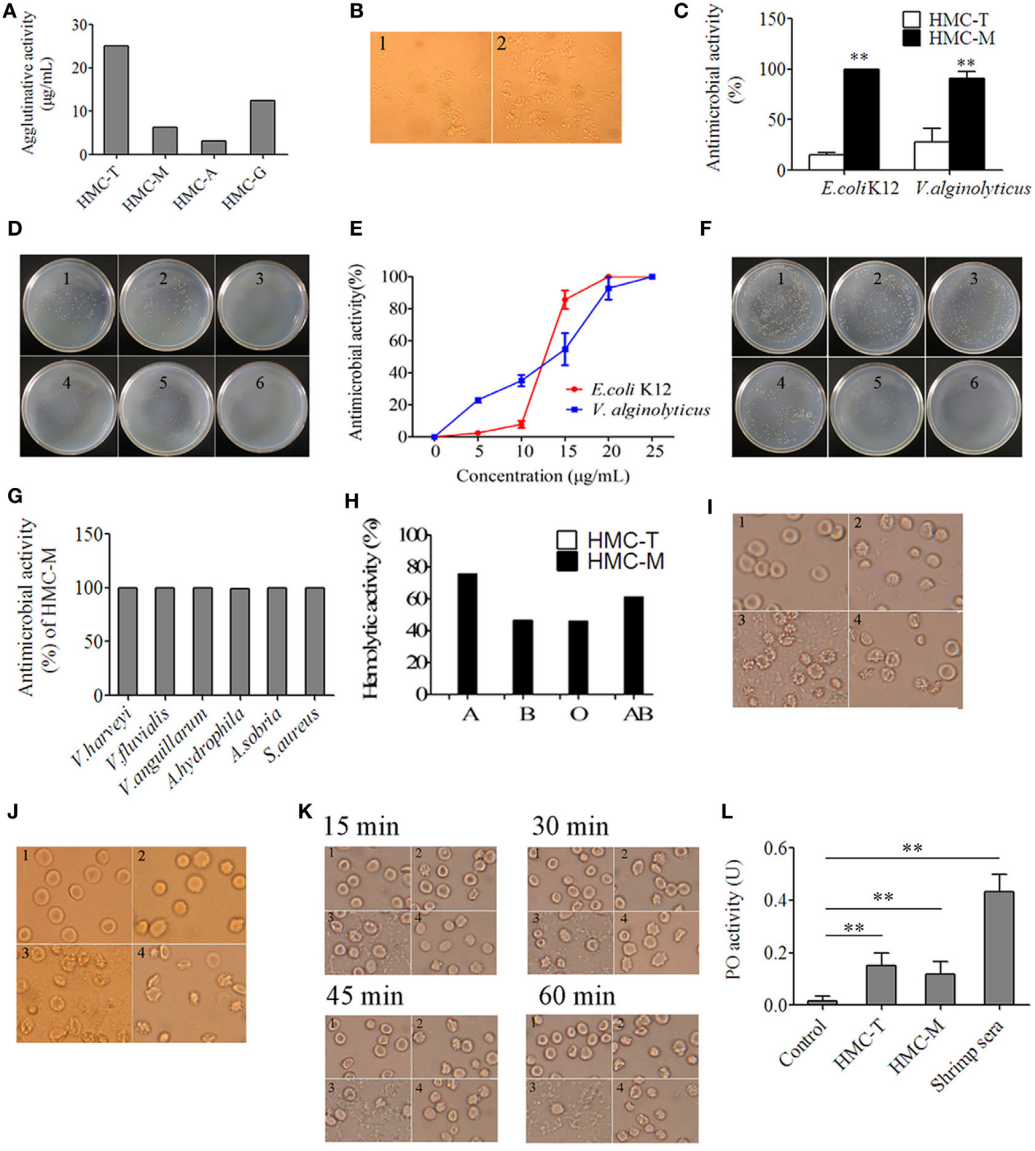
**Diversity of hemocyanin’s (HMC’s) activity**. **(A)** Agglutinative activity between different chromatography purified components of HMC against *Vibrio parahaemolyticus*. **(B)** Microscopic map (1,700×) for agglutinative activity of HMC-M (1) and HMC-T (2) (25 µg/mL) with *V. parahaemolyticus*. **(C)** Antimicrobial activity of HMC-T or HMC-M (20 µg/mL) with *E. coli* K12 or *V. alginolyticus*. Data represent the mean ± SE. of at least three independent experiments, **denotes significant difference relative to control (*p* < 0.01). **(D)** Bacterial colonies in a Petri dish of samples in **(C)**. 1. *E. coli* K12 as negative control; 2. HMC-T + *E. coli* K12; 3. HMC-M + *E. coli* K12. 4. *V. alginolyticus* as negative control; 5. HMC-T + *V. alginolyticus*; 6. HMC-M + *V. alginolyticus*. **(E,F)** Dose-dependent antimicrobial activity of HMC-M against bacteria. Percent survival of *E. coli* K12 and *V. alginolyticus* **(E)**, and bacterial colonies **(F)** in Petri dish of *V. alginolyticus* samples in **(E)**. 1, Negative control; 2–6, 5, 10, 15, 20, and 25 µg/mL of HMC-M were added, respectively. **(G)** Antimicrobial activity of HMC-M (20 µg/mL) from *L. vannamei* against different aquicultural bacteria. **(H)** Hemolytic activity (%) of HMC-T and HMC-M against four types of human erythrocytes. **(I–K)** Hemolytic activity toward human erythrocyte groups A **(I)**, or O **(J)** B and dynamic observation **(K)** at the time indicated point (2,120×). 1. Negative control group; 2. HMC-T; 3. HMC-M; and 4. positive control group (ddH_2_O). **(L)** PO activity between HMC-T and HMC-M. Data represent the mean ± SE. of at least three independent experiments. No significant difference was shown between HMC-T and HMC-M (*p* > 0.05, *p* = 0.46005).

We reckoned that gene diversity should be related to biological function. To demonstrate this, we used anti-human IgM, anti-human IgA, and anti-human IgG to recognize different HMC fractions, namely HMC-M, HMC-A, and HMC-G, respectively. In addition, anti-shrimp HMC raised using purified HMC was used to isolate HMC (HMC-T) as a control. Four assays, i.e., agglutination assay, antimicrobial activity assay, hemolytic activity assay, and phenoloxidase (PO) activity assay, were carried out so as to determine biological function. First, the assay on HMC agglutination with *V. parahaemolyticus* showed that agglutinative activity increased by at least four fold in HMC-M (6.25 µg/mL) compared with HMC-T (25 µg/mL) (Figure 4A). As shown in Figure 4B, bacteria treated with HMC-M had more aggulutination than that treated with HMC-T. Secondly, higher antimicrobial activity was observed in HMC-M than in HMC-T treated samples. Using two bacteria, i.e., a marine pathogen *V. parahaemolyticus* and a model *E. coli* K12, the percentage inhibition by HMC-T and HMC-M were 15.47 ± 1.62 and 99.99 ± 0.11% for *E. coli* K12 (*p* < 0.01), respectively, and 27.76 ± 13.94 and 91.00 ± 6.50% for *V. alginolyticus* (*p* < 0.01), respectively (Figures 4C,D). We further showed that the antimicrobial activity of HMC-M against both of *E. coli* K12 and *V. alginolyticus* increased dose dependently. A dynamic curve showed that *E. coli* K12 was only sensitive to higher than 10 µg/mL dose of HMC-M, while *V. alginolyticus* presented a typical concentration-dependent trend. When 20–25 µg/mL HMC-M was used, the two bacteria were completely inhibited (Figures 4E,F). We further demonstrated the antimicrobial activity of HMC-M in other bacterial pathogens such as *V. harveyi, V. fluvialis, V. anguillarum, A. hydrophila, A. sobria*, and *S. aureus*. Similarly, 20 µg/mL HMC-M completely killed or inhibited these bacteria (Figure 4G). Next, hemolytic assay was performed using human erythrocytes from blood types A, B, O and AB with 200 µg/mL of HMC-T and HMC-M. Only HMC-M showed obvious hemolytic activity with values 72.57 ± 2.62, 46.17 ± 4.58, 45.97 ± 0.96, and 61.10 ± 2.75% for A, B, O, and AB human erythrocytes, respectively (Figure 4H). Figures 4I–K show the representative images of the hemolysis of human type A and O erythrocytes by HMC-M, and the dynamic process of hemolysis of human type B erythrocytes. Finally, the PO activity was compared between HMC-T and HMC-M, with no significant difference observed between HMC-M and HMC-T (Figure 4L). These results indicate that HMC-M possesses strong bacterial agglutination, bacterial growth inhibition and human erythrocyte hemolysis.

### HMC Recognizes Bacteria by Their PAMPs Such as OmpT, OmpW, OmpX, OmpC, OmpA, and FadL

**Figure 5:**
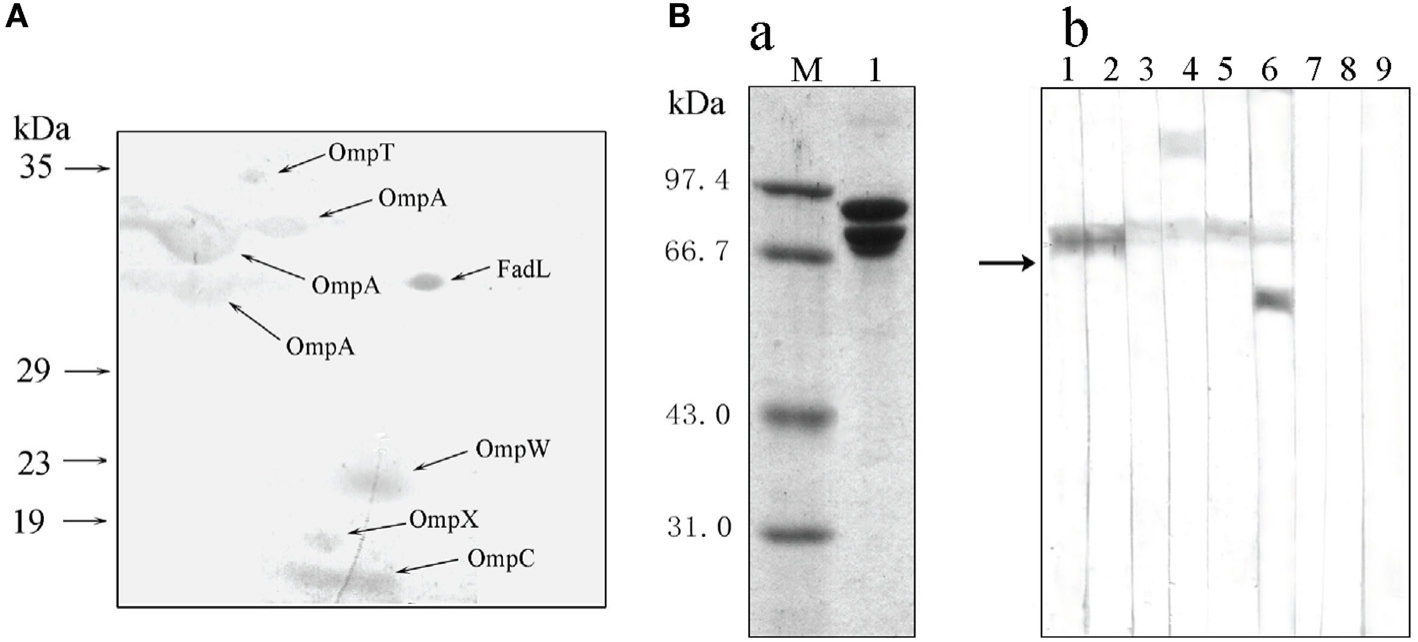
**Bacterial pathogen-associated molecular patterns (PAMPs) recognized by the receptor hemocyanin (HMC)**. **(A)** Identification of bacterial proteins bound to HMC by 2-D Far-Western blot analysis using *E. coli* K12 sarcosin-insoluble fraction as antigens and *L. vannamei* HMC as a bridge. **(B)** Identification of bacterial Omps recognized by HMC. (a) One-dimensional sodium sulfate-polyacrylamide gel electrophoresis (1-DE) analysis of HMC isolated from bacterial pull-down fraction (2). (b) Far-Western blot analysis using HMC as an antigen and *E. coli* K12 sarcosin-insoluble fraction as a bridge, and rabbit antibodies against *E. coli* OmpX (1), OmpW (2), OmpA (3), OmpT (4), OmpC (5), FadL (6), LamB (7), Dps (8), or pre-immunized rabbit sera (9) as primary antibodies.

To investigate the recognition of bacterial PAMPs by the receptor HMC D3, the strategy of protein-protein interaction was used to detect HMC ligands. The outer membrane proteins (Omps) of gram-negative bacteria are involved in the first line of bacterial physiology and pathogenesis (50–53). Therefore, the sarcosine-insoluble fraction of *E. coli* K12 envelope was isolated by 2-DE and transferred onto nitrocellulose membranes for Far-Western blot analysis. The analysis was performed using HMC and anti-shrimp HMC-T antibody, as bridge protein and primary antibody, respectively. Eight spots were stained on the nitrocellulose membranes, with six of them identified as unique proteins. These were OmpT, OmpW, OmpX, OmpC, OmpA (three spots), and FadL (Figure 5A; Table S2 in Supplementary Material). HMC ligand blot assay was also performed to confirm the results obtained from the 2-D Far-Western blot. Here, HMC isolated from the bacterial pull-down fraction was blotted onto nitrocellulose membrane, followed by cutting the membrane into strips, incubation separately with OmpT, OmpW, OmpX, OmpC, OmpA, or FadL, and then with their corresponding antibodies. Meanwhile, antibodies to the Omps, i.e., LamB or Dps, were used as negative controls. Our results showed that the six antibodies could react with their corresponding antigens bound with HMC on the nitrocellulose membrane but not with the control antibodies (Figure 5B). These results further suggest that the six antigens of OmpT, OmpW, OmpX, OmpC, OmpA, and FadL were ligands for the receptor HMC.

### Functional Diversity of HMC-M Was Determined Using Genetically Modified Strains, Which Had HMC Ligands Genes Deleted from Bacterial OM

**Figure 6:**
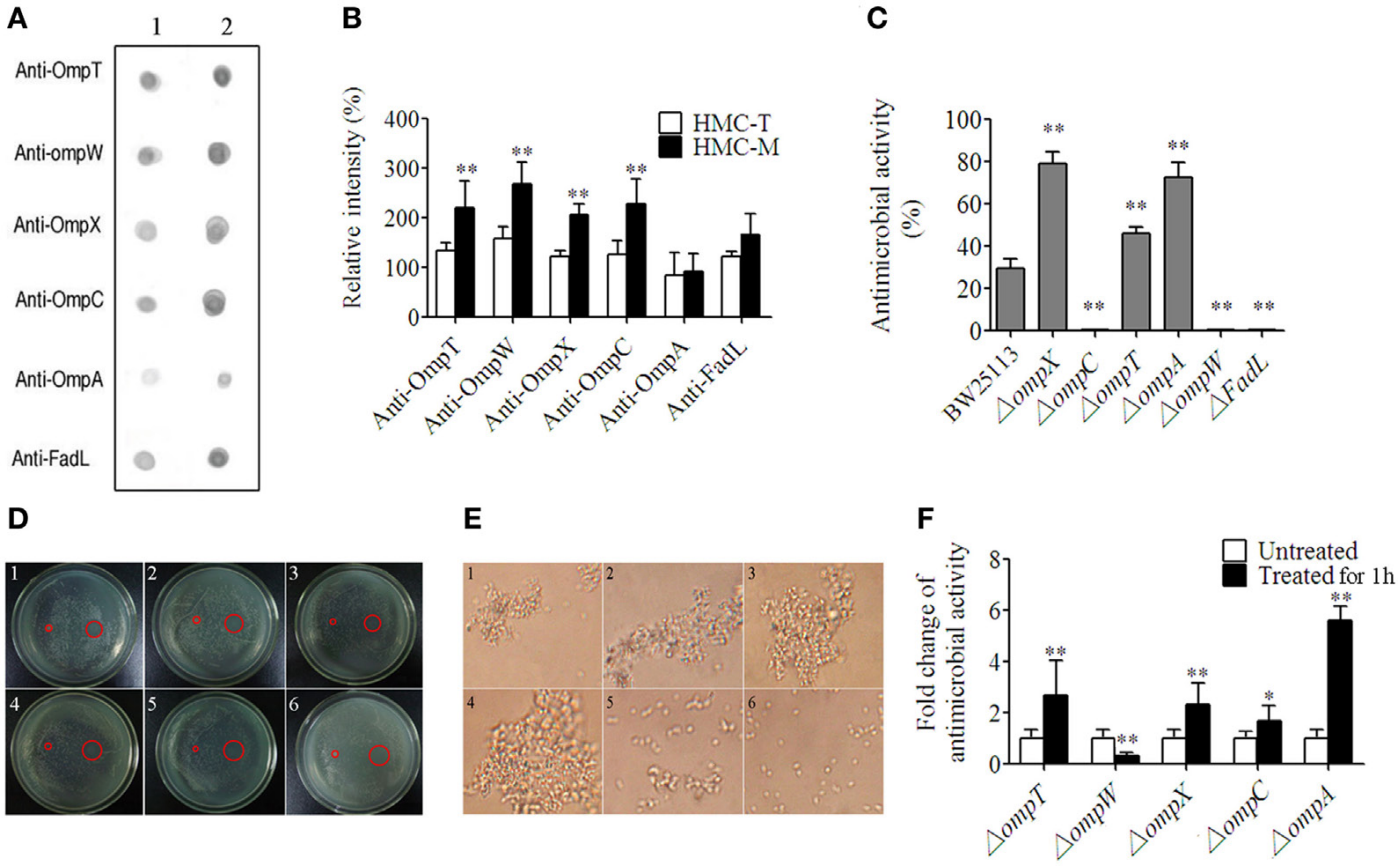
**Diversity of hemocyanin (HMC) recognition and inhibition of bacteria**. **(A)** Far-Dot-ELISA analysis using HMC-T (1) and HMC-M (2) as antigens, and OmpT, OmpW, OmpX, OmpC, OmpA, and FadL as bridges. **(B)** Histogram showing the changes in spot intensity. **(C,D)** Antimicrobial activity of HMC-M against genetically modified outer membrane proteins gene-deletion strains. **(C)** 12 µg/mL of HMC-M by using plate count method. These data represent the mean ± SE. of at least three independent experiments. *p < 0.05, **p < 0.01 are the significant difference compared with control. **(D)** 200 µg/mL of HMC-M (right) and HMC-T (left) using zone inhibition assay. 1–6. 50 µL of genetically modified *E. coli* K12 BW25113 strains (10^7^–10^8^ CFU/mL) with the gene deletion of OmpT, OmpW, OmpX, OmpC, OmpA, and FadL, respectively. **(E)** Micrograph (4,400×) for agglutinative activity of *E. coli* K12 with HMC fraction purified by (1) OmpT-, (2) OmpX-, (3) OmpC-, (4) OmpA-, and (5) DpS-chromatography or (6) HMC-T. **(F)** Antimicrobial activity analysis of the sera from *L. vannamei* treated with *E. coli* K12 BW25113 and its different mutants *in vivo*.

As described earlier, the *E. coli*, OmpT, OmpW, OmpX, OmpC, OmpA, and FadL were recognized by HMC. Thus, we went on to perform the functional characterization of HMC-M in response to these six ligands. First, we characterized the differences between the binding of these Omps with HMC-M and HMC-T as well as the ability of HMC-M in binding the ligands using Far-Dot-ELISA assay. We observed high intensity of the spots for OmpT, OmpC, FadL, OmpW, and OmpX but not OmpA, for HMC-M with respect to HMC-T as a blot antigen. Moreover, there were differences in the binding ability to the six Omps between HMC-M and HMC-T. The observed binding ability from high to low is as follows: OmpW > OmpC and OmpT > OmpX > FadL > OmpA (Figures 6A,B). These results indicate that HMC-M is a key component in binding OM ligands, with this binding ability being ligand dependent. Next, we sought to find out whether the binding was responsible for the antibacterial activity, by using genetically modified strains in which the genes for these six ligands are deleted. Using a concentration of 12 µg/mL for HMC-M against *E. coli* K12 BW25113, as well as the parent strain of these gene-deleted mutants as a control, we observed that deletion of *ompX, ompA*, or *ompT* resulted in an elevation in the antimicrobial action, i.e., 79.08 ± 5.36, 72.46 ± 7.35, and 46.15 ± 3.04%, respectively, relative to control (29.46 ± 4.64%). On the other hand, the absence of *ompC, ompW*, or *fadL* resulted in a decrease in the bacterial growth inhibition (Figure 6C). Given our observations that HMC was the major protein in shrimp sera, which bound with bacteria and that HMC-M possessed strong immune functions, the *in vivo* antimicrobial activity of shrimp sera against *E. coli* K12 BW25113 (ampicillin resistance) and the gene-deleted mutants (kanamycin resistance) was further investigated. As anticipated, except for the Δ*ompC* mutant, the results were similar to that observed in the *in vitro* antimicrobial analysis for HMC-M. The rate of inhibition of shrimp sera against the deletion mutants of *ompX, ompA*, or *ompT* was higher than that of the control, while the *ompW* deletion mutant resulted in a decrease in the bacterial growth inhibition (Figure 6D). Collectively, these results suggest that the recognition of bacteria by the innate immune defense molecule, HMC, does not always contribute to bacterial growth inhibition, couple with the suggestion that the bacterial growth inhibition ability of HMC-M depends on other factors so as to exert an effect on multiple targets. Further studies ought to be carried out so as to fully understand this intricate network mechanism. In addition, the bacterial growth inhibition using 200 µg/mL of HMC-M and HMC-T against these mutants was also investigated by the method of zone inhibition assay. As shown in Figure 6E, there was obvious inhibition zone induced by HMC-M against all the mutants, with the diameter of the inhibition zones ranging from 1.23 ± 0.06 to 1.50 ± 0.10 cm, while no bacterial growth inhibition was observed for HMC-T under the same conditions, suggesting that the antibacterial activity of HMC-M may also be concentration-dependent. Probing further, we isolated HMC components bound with OmpT, OmpX, OmpC, OmpA, and Dps (Dps was used as negative control) by chromatograph, and compared their ability to agglutinate *E. coli* K12 BW25113. We observed that the agglutinative activity of the HMC components bound with OmpA, OmpC, OmpT, OmpX, and HMC-T was 0.25, 0.03125, 0.0625, 0.0625, and 12.5 µg/mL, respectively (Figure 6F). These agglutinative activities of the components were 50–400 higher than that of HMC-T. Interestingly, the agglutinative ability is in the order OmpC > OmpT and OmpX > OmpA, which is basically consistent with the difference in binding between the Omps with HMC-M and HMC-T. Therefore these results strongly indicate the diversity of HMC components.

### Protein and Glycosylation Differences between HMC-M and HMC-T Might Contribute to the Functional Diversity of HMC

**Figure 7:**
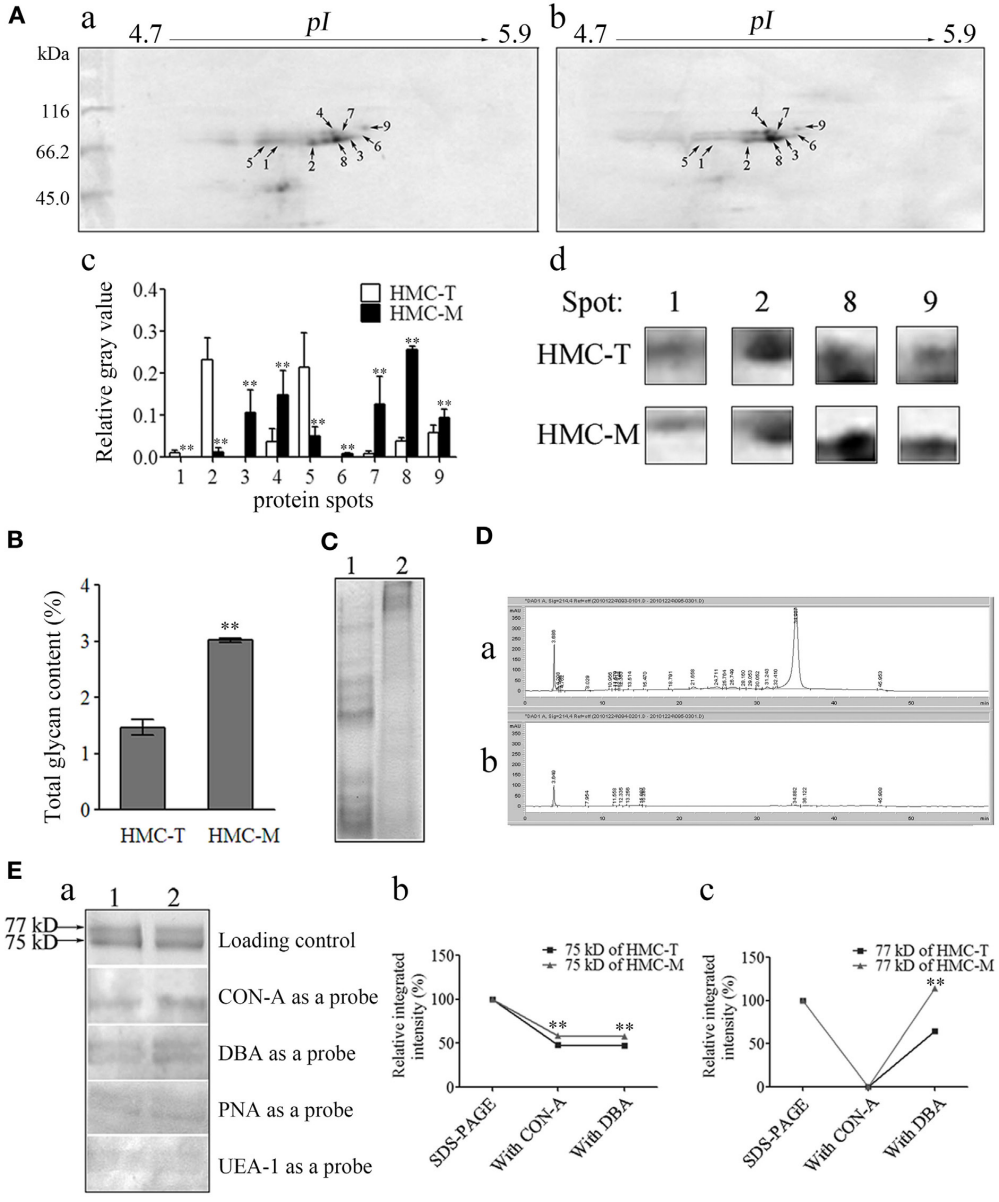

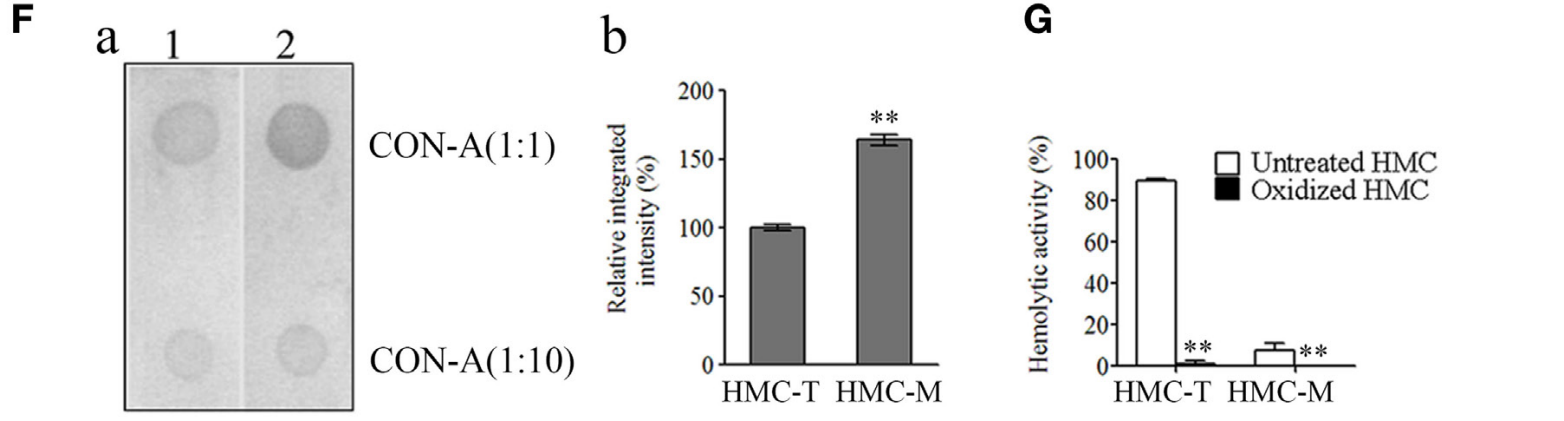
**Diversity of protein and glycan modification of hemocyanin (HMC)**. **(A)** Two-dimensional polyacrylamide gel electrophoresis (2-DE) map for shrimp HMC identified by MALDI-TOF-TOF mass spectrometer and recognized by anti-shrimp HMC-T (a) and HMC-M (b). (c) Bar graph showing the gray value of differentially expressed proteins. Bars represent mean ± SD. of normalized spot intensity determined using the PDQuest software version 8.0 (Bio-Rad, CA, USA, *n* = 3). (d) Highlight of partial differentially expressed protein spots. **(B)** Glycan concentration of HMC-M compared to HMC-T using the method of phenol-sulfate acid. Data represent the mean ± SE. of at least three independent experiments, **p < 0.01. 1, HMC-T; 2, HMC-M. **(C–F)** Glycan components between HMC-T and HMC-M. Tricine-SDS-PAGE **(C)** and high-performance liquid chromatography (HPLC) analysis **(D)**. (1)/(a), HMC-T; (2)/(b), HMC-M. Lectin blot analysis using biotinylated lectin **(E)**. (a) One-dimensional sodium sulfate-polyacrylamide gel electrophoresis (1-DE) for lectin blot analysis. Concanavalin A (CON-A), dolichos biflorus agglutinin (DBA), peanut agglutinin (PNA), or ulex europaeus agglutinin 1 (UEA-1) (1:5) and peroxidase-conjugated avidin (1:1,000). (b and c) Relative integrated intensity of HMC-T and HMC-M. **(F)** Far-Dot-ELISA analysis. (a) Far-Dot-ELISA using HMC-T (1) or HMC-M (2) as a bridge, and rabbit anti-shrimp HMC-T antisera and goat anti-rabbit IgG-HRP (1:3,000) antibodies used as primary and second antibodies, respectively. (b) Relative integrated intensity of dot (1:1 of CON-A) between HMC-T and HMC-M. **(G)** Hemolytic activity between MHC-T and MHC-M with or without carbohydrate epitopes. These data represent the mean ± SD of at least three independent experiments, **p < 0.01.

To investigate the molecular basis for the functional diversity of HMC, comparative proteomics was used. Following 2-DE, 11 and 10 spots on HMC-T and HMC-M gels were distinguished by PDQuest software, with sizes mainly around 75 and 77 kDa. Nine protein spots on the HMC-M gel were significantly altered compared with the HMC-T gel. Of these, proteins at spots 3–4, 6–9 were upregulated, while proteins at spots 1, 2, 5 were downregulated (Figure 7A). All the 21 spots from the HMC-M and HMC-T gels were excised and subjected to MALDI-TOF-TOF mass spectrometer analysis. As anticipated, the 21 protein spots were identified as either *L. vannamei* S-HMC or L-HMC (data not shown). Protein spots with unique pI but identified as the same protein on a 2-DE gel are said to possess non-synonymous SNPs (54, 55). As shown in a previous section that, the D3 domain contained several hot spots of SNPs, which together with the 2-DE results seems to suggest that the diversity of HMC at the protein level might be due to the SNPs.

During the mass spectrometry analysis, we noticed that some peptides showed mass shifts, which may be due to post-translational modifications (56, 57). Since glycosylation is one of the most important post-translational modifications of Ig to regulate its function (58), we hypothesize that probably HMC might also be glycosylated, especially HMC-M to regulate its functions. We therefore went about to compare the glycosylation of HMC-M and HMC-T by measuring the total amount of glycan, glycan components, and glycan oxidization. To begin with, total glycan concentration was measured using the phenol-sulfate acid method. A higher glycan concentration was found in HMC-M (3.023 ± 0.0523%) than in HMC-T (1.468 ± 0.1923%) (Figure 7B). Next, the difference in glycan components between HMC-M and HMC-T was determined using Tricine-SDS-PAGE, HPLC, lectin blot, and Far-Western blot analysis. The results from the Tricine-SDS-PAGE analysis revealed that the molecular weights of the main degraded fragments digested with trypsin from HMC-M were significantly higher than those from HMC-T (Figure 7C). These degraded peptides were further analyzed by HPLC. Approximately 10 and 24 peaks were observed in HMC-M and HMC-T, respectively. In particular, 67.6 and 8.6% peptides from HMC-M and HMC-T were detected at 3.6 min, while 8.4 and 76.1% were found at 34.9 min (Figure 7D). Consistent with the above observations from total glycan concentration determination, these findings suggested that HMC-M had higher glycosylation compared to HMC-T. Moreover, lectins including CON-A (recognizing α-d-mannose > α-d-glucose), DBA (recognizing *N*-acetyl-d-galactosamine), PNA (recognizing β-d-gal-(1,3)-d-acetyl galactosamine), and UEA-1 (recognizing α-l-fucose) were used to blot the type of glycans in HMC-T and HMC-M. We observed that CON-A only bound the small subunit of both HMC-T and HMC-M, while the other three lectins could recognize the two subunits of them in a degree. Notably, both CON-A and DBA had more extensive staining with HMC-M than HMC-T, on the contrary, no difference between HMC-M and HMC-T with the PNA and UEA staining was found. This data indicates that HMC-M contains more α-d-mannose > α-d-glucose and *N*-acetyl-d-galactosamine than HMC-T (1.21:1 and 1.54:1, respectively, in CON-A and DBA) (Figure 7E). Consistent with the Far-Dot-ELISA results, the relative integrated intensity in HMC-M was about 1.6-fold that of HMC-T. To further substantiate the glycosylation of HMC-M by CON-A and DBA, HMC-T and HMC-M were incubated with blotted lectin CON-A, and then reacted with anti-shrimp HMC-T antibodies (Figure 7F). It was observed that there was a reduction in α-d-mannose > α-d-glucose but an increase in *N*-acetyl-d-galactosamine in HMC-M. We then went on to investigate the effects of glycan-free HMC on hemolytic activity. Both HMC-M and HMC-T were first subjected to the periodate oxidation assay, which results in the destruction of their carbohydrate epitopes. Interestingly, the hemolytic activity was completely abolished after deglycoslyation by oxidization, as there was a drop from 89.41 to 0.93% for HMC-M and from 7.53 to 0% for HMC-T (Figure 7G). Thus, with these results on the glycosylation of HMC-M couple with the SNPs in the D3 domain it can be speculated that all these together might contribute to the functional diversity of HMC.

## Discussion

Unlike vertebrates, which have both innate and adaptive immunity, invertebrates only have innate immunity that is constituted by immune-like molecules such as Toll receptor, Dscam, FREP, etc. The question that is often raised is, how do invertebrates deal with various types of infections using such an unsophisticated immune system composed of only a limited number of molecules. Therefore, unearthing the mechanisms of action of these molecules will provide novel insights, which would enable us better understand innate immunity from an evolutionary perspective. Progress has been made in recent years, with reports on the identification of homology of critical complement components and the opsonic defense mechanism in the Precambrian ancestor of bilateral animals (the horseshoe crab) and a cephalochordate of a basal lineage of chordate (amphioxus) (18, 59, 60). Similarly, novel innate defense molecules which possess extensive polymorphism and may be related to primary adaptive immunity have been found in invertebrates including FREP3 in snails, Dscam in *Drosophila*, V region-containing chitin-binding proteins in *Branchiostoma floridae* and 185/333 gene cluster in the purple sea urchin *Strongylocentrotus purpuratus* (3, 19, 61, 62). Several reports have indicated the importance and capability of HMC in destroying bacteria, viruses, fungi, inhomogeneous erythrocytes, and tumors (20–27, 63, 64). A systemic study by Ding et al. reported that HMC was directly activated by microbial proteases and was enhanced by PAMPs as a frontline defense molecule in horseshoe crab (18, 20). In accordance with Ding et al.’s observation, which showed that at least several proteins were involved in the frontline immunity recognition in horseshoe crab (18), our results indicates that HMC was also the sole major innate defense protein against invading bacteria in shrimp. Given that both horseshoe crab and shrimps are crustaceans, it thus suggests the diversity of frontline defense molecules exists in invertebrates.

Recent reports suggest that molecules with innate immune functions may not directly and/or structurally share similar predecessors with adaptive immunity molecules, implying the boundary between adaptive and innate immunity is no longer clear cut as has been the notion for decades (65–67). For instance, the full-length sequence of HMC is not directly matched or share significant similarity with any immune molecules using conventional alignment tools, except for prophenoloxidase. However, HMC D3 may be annotated as a member of the Ig-like superfamily. Despite this, little is known about any similar adaptive immunity molecule successor of the domain. Here, we present some novel findings that HMC D3 is a homolog of the variable regions of human Ig in terms of both sequence and three-dimensional structure, and also of the sequences of defense molecules in *Drosophila* and *C. elegans*. The HMC D3 domain shares same or similar epitopes with those in human Ig variable regions, and thus can cross-react with anti-human Ig, while HMC D1 and D2 cannot and therefore are not involved in the primordial form of immunosurveillance. Furthermore, HMC D3 possesses extensive SNPs, independent of tissue, individual and developmental stage of shrimp, just as in human Ig variable regions. It therefore suffice to say, the similarity between HMC and natural antibodies are (1) both are the major proteins in the circulatory system; (2) both are cross reactive; (3) similar epitope sequences and spatial structure are shared by both HMC D3 and Ig variable regions; (4) both display unexpected SNPs; and (5) both react to microbes. Hence, HMC-M may probably be the evolutionary precursor of Ig and its D3 domain (Ig-like domain) might be responsible for the antibacterial and hemolytic activities.

Several reports have indicated that HMC, a respiratory protein, may also function in innate immune defense against viruses, bacteria and fungi in arthropod and mollusk (68–70). It has been shown that HMC could be activated and functionally converted into a phenoloxidase-like enzyme by reagents including trypsin, α-chymotrypsin, percholate, and SDS (71–74), as well as by endogenous biodefence molecules such as clotting factors and antibacterial peptides (21, 75), and even by hemocyte lysate supernatant (76). The conversion of HMC is enhanced by PAMPs and results in the production of highly reactive cytotoxic quinones, which effectively kills microbial intruders (20). Meanwhile, it has been demonstrated that this protein could generate C-terminal fragments, which have broad antifungal and antibacterial activities (22, 23). Here, we have revealed a previously unknown recognition mechanism of HMC to bacteria. Instead of intracellular and bacterial serine proteases and/or antimicrobial peptides, we have provided evidence of a direct and acute frontline antimicrobial action of HMC through binding of *E. coli* PAMPs, i.e., OmpT, OmpW, OmpX, OmpC, OmpA, and FadL. Moreover, we have shown that this binding can result in diverse biological actions including bacterial agglutination and growth inhibition, as well as human erythrocytes hemagglutination and hemolysis. Equally important in this finding is the fact that, this non-TLR innate immune molecule (HMC) plays a role in antimicrobial action by sensing six PAMPs (77–79). Among these PAMPs, we observed that OmpC, FadL, and OmpW were targets of HMC-M as the antibacterial effectors, while OmpT, OmpA, and OmpX seems to negatively regulate this action. Unlike HMC-M, which recognizes several *E. coli* PAMPs, SAA in human plasma mainly recognizes *E. coli* OmpA (78, 80). Thus, HMC-M can recognize more PAMPs than SAA, and the recognitions of HMC and SAA to OmpA result in reverse downstream effects. More work is required to further unravel the differences in the recognition mechanisms of PAMPs by invertebrates and vertebrates. While we cannot conclude that the differences may be associated with evolution, our current results indicate that the targeting of multiple molecules is a characteristic feature of HMC-M in its antimicrobial action.

Molecular diversity is a characteristic feature of adaptive immunity, with well documented and extensive studies on the diversity of Ig, TCR, and MHC. It is reported that diverse repertoire of TCR and Ig molecules encoded by a diverse of multiple gene families with similar but non-identical sequences can specifically recognize “self” from “foreign” (80). Unlike adaptive immunity, innate immunity recognizes PAMPs using PRRs. The molecular diversity of PRRs has been shown in several studies (3, 19, 61, 62), however, the types and mechanisms of this diversity need to be further examined. Here, we have revealed a previously unknown recognition mechanism, in that HMC-M contributes to a diversified reorganization and the frontline antimicrobial action by extensive diversities at the level of nucleic acid sequence, protein expression and glycosylation modification. Of important note are the changes in the glycan composition of HMC-M including a significant increase in α-d-mannose > α-d-glucose and *N*-acetyl-d-galactosamine.

In conclusion, we have shown that HMC is a major innate non-TLR protein in *L. vannamei*. The the C-terminal domain of HMC has a homologous sequence and structure with the variable region of human Ig, and recognizes the *E. coli* PAMPs OmpT, OmpW, OmpX, OmpC, OmpA, and FadL. In addition, HMC has molecular polymorphisms at the nucleic acid sequence level, as well as protein expression and glycosylation modification, which might synergistically contribute to its functional diversity. Finally, HMC-M, a fraction of HMC, shows strong antibacteria activity and hemolysis, with the strong suggestion that its antimicrobial action is broad spectrum.

## Ethics Statement

This study was carried out in accordance with the recommendations of in the Guide for the Care and Use of Laboratory Animals of the National Institutes of Health of guidelines, name of committee. The protocol was approved by the Institutional Animal Care and Use Committee of Sun Yat-sen University (Animal Welfare Assurance Number: I6).

## Author Contributions

XP, BP, and YZ wrote the manuscript. XP, BP, and YZ conceptualized and designed the project. XP, YZ, BP, HL, and YL interpreted the data. XP, YZ, and BP performed data analysis. YZ, HW, HL, BP, FY, XZ, XL, SM, YG, SW, and BP performed experiments and collected samples.

## Conflict of Interest Statement

The authors declare that the research was conducted in the absence of any commercial or financial relationships that could be construed as a potential conflict of interest.
