# Supplementary material for: C-Terminal Domain of Hemocyanin, a Major Antimicrobial Protein from Litopenaeus vannamei: Structural Homology with Immunoglobulins and Molecular Diversity
Source: Front Immunol. 2017 Jun 13;8:611. doi: 10.3389/fimmu.2017.00611 (PMC5468459; doi:10.3389/fimmu.2017.00611)
Supplement: Supplementary file 1 [file image_1.pdf]

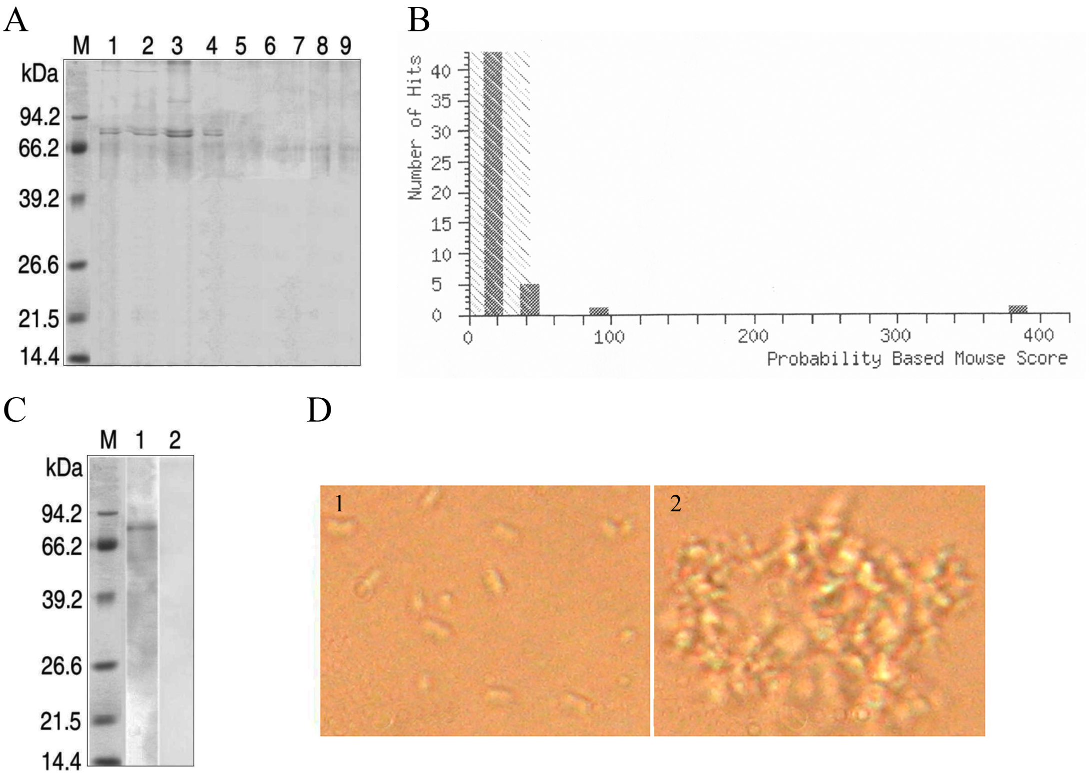

Figure S1. Isolation and characterization of frontline bacteria-binding proteins. **(A)** 1-DE analysis of the bacteria-binding proteins. M, Protein markers; 1-4: Samples respectively from *V. parahaemolyticus, V. fluvialis, P. fluorescens and E. coli* K12 cells incubated with shrimp plasma. 5-9: Samples from *V. parahaemolyticus, V. fluvialis, P. fluorescens and E. coli* K12 cells incubated the 0.01 M phosphate-buffered saline (PBS; pH 7.4) as negative controls. **(B)** The representative Mascot search score of the isolated band bound with *V. parahaemolyticus* by ESI-MS/MS analysis. Score is –10 Log (P), where P is the probability that the observed match is a random event. Individual ions scores>43 indicate identity or extensive homology (p<0.05). The score of HMC (hemocyanin from *Litopenaeus vannamei*) [gi| 854403] is 384. **(C)** Western-blotting analysis of the isolated HMC bound with *V. parahaemolyticus* cells. Lane 1: Protein markers; Lane 2:

Western-blotting analysis of the isolated HMC using rabbit anti-shrimp HMC-T antisera (1:500) and goat anti-rabbit IgG-HRP (1:1000) as the primary and secondary antibodies, respectively ; Lane 3: Western-blotting analysis of the negative control.

(D) Agglutinative activity analysis of the HMC (10 μg/ml) isolated from *V. fluvialis* incubated with shrimp plasma. 1: Negative control group of agglutinative reactivity in *V. fluvialis* (4400×); 2: The agglutinative reactivity of HMC in *V. fluvialis* (4400×).
