# Supplementary material for: C-Terminal Domain of Hemocyanin, a Major Antimicrobial Protein from Litopenaeus vannamei: Structural Homology with Immunoglobulins and Molecular Diversity
Source: Front Immunol. 2017 Jun 13;8:611. doi: 10.3389/fimmu.2017.00611 (PMC5468459; doi:10.3389/fimmu.2017.00611)
Supplement: Supplementary file 2 [file image_2.pdf]

A

```
S-HMC D3   413   PPYTKADLEFSGVSVTELAVVGELETYFEDFEYSL---INAVDDAEGIPDVEISTYVP---- 467
Dm-PP     1088   --------EYQATSPTESSIANEFATAALRFGHTI---INPI-------------------- 1118

S-HMC D3   468   RLNHKEFTFRID---VENGGAERLATVRIFAWPHKDNNGIEYTFDEGRWNAIELDKFWVS-- 524
Hs-SABILL  102   RRDNAAYFFRLKSKWMKYGYTSSKLSVRVMALTHRPNISIPGTLESGHPSNLTCSVPWVC-- 161
Hs-MHC      13   --------------------ADHVSTYAMFVQTHRPTGEFMFEFDEDEMFYVDLDK------ 48
Hs-IgH1     98   -----QFSLKLS---SVTAADTACITVREIWWGYGDNDGMD--------------------- 130
Hs-IgH2     57   ---------------------------------------------------DKFYVD-- 62

S-HMC D3   525   LKG----------GKTSIERKSTESSV-TVPDVPS----IHDLFAEAEAGGAGLAKFES--- 568
Hs-SABILL  162   EQGTPPIFSWMSAAPTSLGPRTTQSSVLTITPRPQ----DH--------------------- 198
Hs-IgL1     53   ---------------------------------------------------------FKR--- 55
Hs-IgH2     63   SVK----------GRFTISRDNAENSV ---------------------------------- 79

S-HMC D3  569   ATGLPNRFLLPKGNDRGLEFDLVVAVTDGDADSA----VPNLHENTEY--NHYGSHG-VYPD 623
Ce-T      1497   --GAPIEAFVIEKKDKNGRWEEALVVPGDQKTAT----VPNLKEGEEY-------------- 1538
Dm-DPTP    900   ---LPNRF-----SDR --------------------------------------------- 907
Dm-DLAR    133   -------------------------------------------------------------D 133
Hs-CD99     43   -------- IPKKPSAGDDFDLGDAVVDGENDDPRPPNPPKPMPNPNP--NHPSSSG-SFSD 92
Hs-IgL1     56   ATGIPDRF---SASGSGTDFALTIS ----------------------------------  77
Hs-IgL2     48   ATGIPNRF---SGSGSGTDFTLTISRVEPE-DSA----V--------YYCQHYGS------  86
Hs-IgH3     41   ---------------------------------------------EI--NHYGSRN-YNPS 53
```

B

```
Igκ  (gi|722524)         48 ATGIPNRF---SGSGSGTDFTLTISRVEPE-DSAVYYCQ------HYGS 86
[H. sapiens]
S-HMC (gi|854403)       569 ATGLPNRFLLPKGNDRGLEFDLVVAVTDGDADSAVPNLHENTEYNHYGS 617
[L. vannamei]
L-HMC (gi|7414468)      577 ATGIPNRFLIPKGNEQGLEFDLVVAVTDGAADAAVDGLHENTEFNHYGS 625
[L. vannamei]
HMC(gi|16612121)        354 ATGIPNRFLLPKGNEQGLEFDLVVAVTDGAADAAVDGLHENTEFNHYGS 402
[P. monodon ]
```

C

```
L-HMC:                                572   KDFESATGIPNRF 584
(gi|7414468) [L. vannamei]                  KD E A+G+P+RF
IgL-VR:                                68   KDTERASGVPDRF 80
(gi|22095259) [H. sapiens]
L-HMC:                                572   KDFESATGIPNRF 584
(gi|7414468) [L. vannamei]                  KD E A+GIP RF
Igλ-VR:                                49   KDNERASGIPERF 61
(gi|3143574) [H. sapiens]
L-HMC:                                574   FESATGIPNRF 584
(gi|7414468) [L. vannamei]                  F  ATGIP+RF
Igκ :                                  45   FSRATGIPDRF 55
(gi|722486) [H. sapiens]
```

Figure S2. Amino acid sequence alignment of HMC D3 domain. (A) Representative alignment of S-HMC D3 with homologous fragments of other species. S-HMC D3, Ig-like domain of *L. vannamei* hemocyanin (gi|854403); Ce-T, *Caenorhabditis elegans* twitchin (gi|6898); Dm-DPTP, *Drosophila melanogaster* protein tyrosine phosphatase (DPTP) precursor (gi|158189); Dm-DLAR, *D. melanogaster* protein tyrosine phosphatase DLAR (gi|1209648); Dm-PP, *D. melanogaster* peroxidasin precursor (gi|531385); Hs-SABILL, *H. sapiens* sialic acid binding Ig-like lectin 6 (gi|20258598); Hs-CD99, *H. sapiens* T-cell surface glycoprotein E2 precursor (E2 antigen) (CD99 antigen) (MIC2 protein) (12E7) (gi|119049); Hs-MHC, *H. sapiens* MHC class II antigen (gi|3757788); Hs-IgL1, *H. sapiens* Ig light chain variable region (CAB46464); Hs-IgL2, *H. sapiens* immunoglobulin light chain variable region (CAB46464); Hs-IgH1, *H. sapiens* Ig mu

heavy chain variable region (gi|4995350); Hs-IgH2, *H. sapiens* IgM heavy chain variable region, VH3 family (gi|33318922); Hs-IgH3, *H. sapiens* IgA heavy chain (gi|7161007). (**B**) Alignments of different types of HMC D3 (gi|854403; gi|7414468; gi|16612121) with Igκ (*H. sapiens* immunoglobulin kappa chain, gi|722524). (**C**) Representative alignments of L-HMC (gi|7414468) with different human Ig light chains. IgL-VR: Immuoglobulin light chain variable region (gi|22095259); Igλ-VR: Immuoglobulin lambda light chain variable region (gi|3143574); Igλ: Immuoglobulin kappa light chain (gi|722486).
