# Supplementary material for: C-Terminal Domain of Hemocyanin, a Major Antimicrobial Protein from Litopenaeus vannamei: Structural Homology with Immunoglobulins and Molecular Diversity
Source: Front Immunol. 2017 Jun 13;8:611. doi: 10.3389/fimmu.2017.00611 (PMC5468459; doi:10.3389/fimmu.2017.00611)
Supplement: Supplementary file 3 [file image_3.pdf]

A

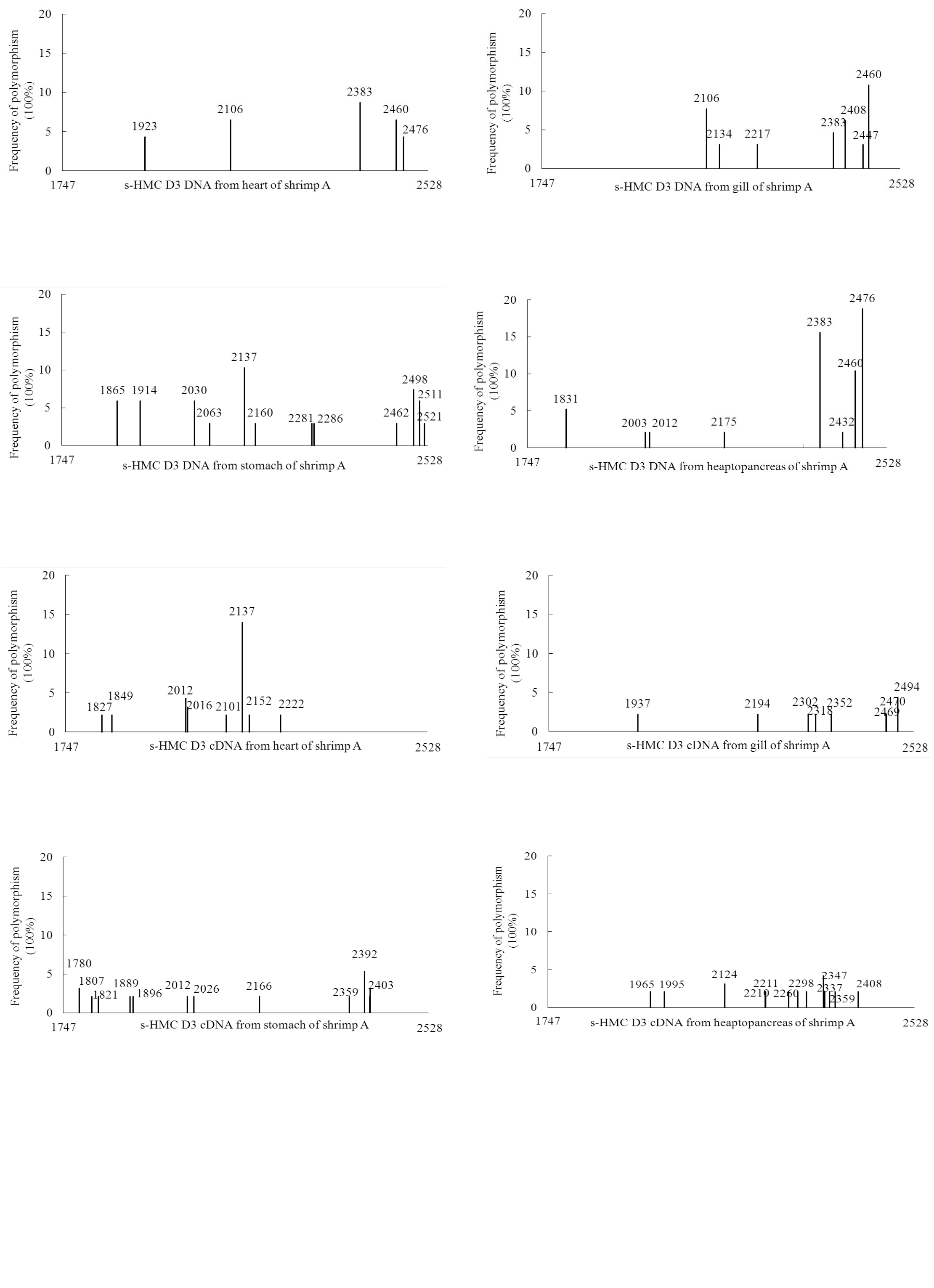
Frequency of polymorphism (100%)
s-HMC D3 DNA from heart of shrimp A
s-HMC D3 DNA from gill of shrimp A
s-HMC D3 DNA from stomach of shrimp A
s-HMC D3 DNA from heaptopancreas of shrimp A
s-HMC D3 cDNA from heart of shrimp A
s-HMC D3 cDNA from gill of shrimp A
s-HMC D3 cDNA from stomach of shrimp A
s-HMC D3 cDNA from heaptopancreas of shrimp A
1747
2528
1923 2106 2383 2460 2476
2106 2134 2217 2383 2408 2460 2447
1865 1914 2030 2063 2137 2160 2281 2286 2462 2498 2511 2521
1831 2003 2012 2175 2383 2476 2460 2432
1827 1849 2012 2016 2101 2137 2152 2222
1937 2194 2302 2318 2352 2469 2470 2494
1780 1807 1821 1889 1896 2012 2026 2166 2359 2392 2403
1965 1995 2124 2210 2211 2260 2298 2337 2347 2359 2408

B

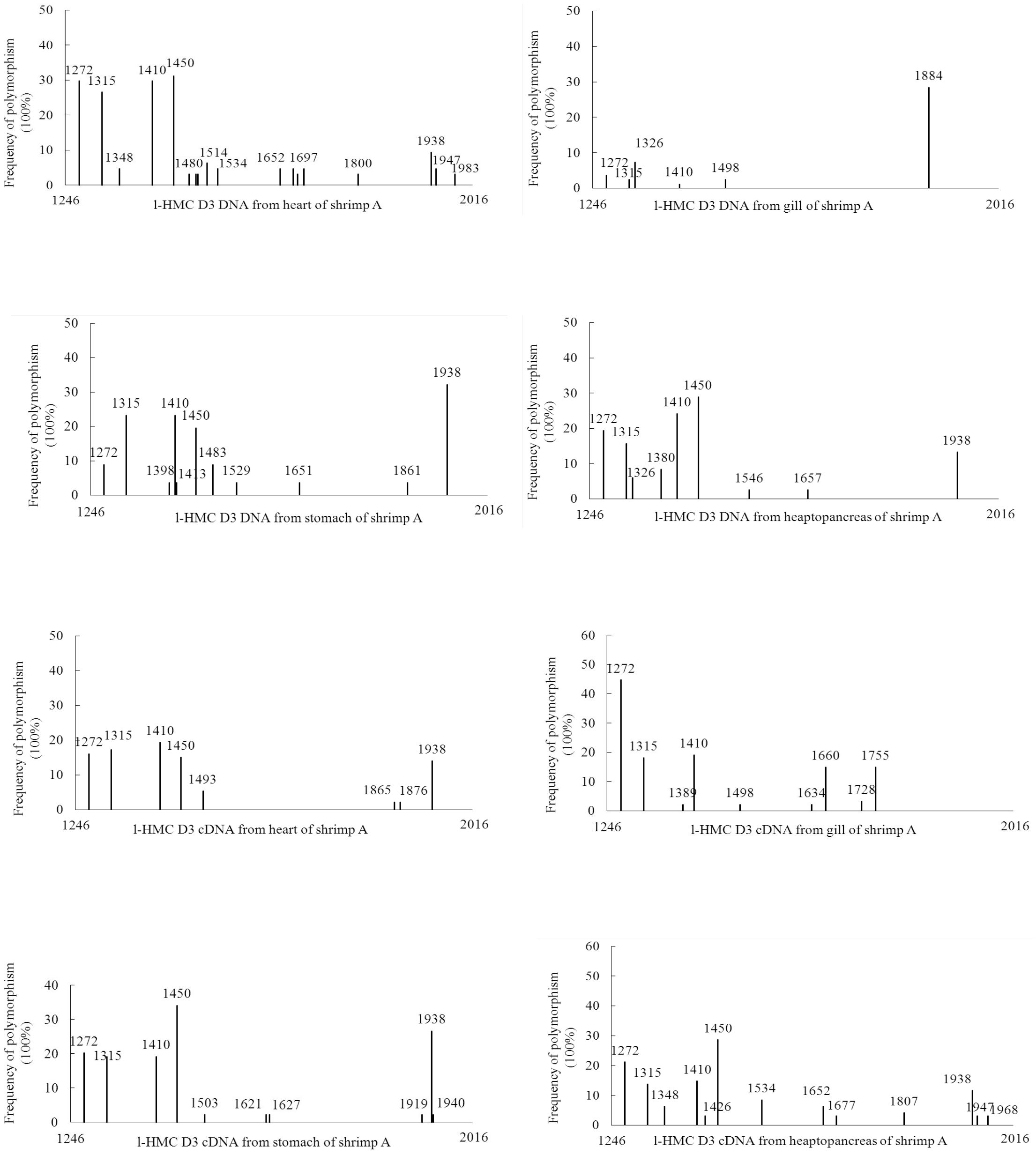
Frequency of polymorphism (100%)
1272
1315
1348
1410
1450
1480
1514
1534
1652
1697
1800
1938
1947
1983
1246
2016
l-HMC D3 DNA from heart of shrimp A
1884
1272
1315
1326
1410
1498
1246
2016
l-HMC D3 DNA from gill of shrimp A
1938
1315
1410
1450
1272
1483
1398
1413
1529
1651
1861
1246
2016
l-HMC D3 DNA from stomach of shrimp A
1450
1410
1272
1315
1380
1326
1938
1546
1657
1246
2016
l-HMC D3 DNA from heaptopancreas of shrimp A
1272
1315
1410
1450
1493
1938
1865
1876
1246
2016
l-HMC D3 cDNA from heart of shrimp A
1272
1315
1410
1389
1498
1660
1755
1634
1728
1246
2016
l-HMC D3 cDNA from gill of shrimp A
1450
1938
1272
1315
1410
1503
1621
1627
1919
1940
1246
2016
l-HMC D3 cDNA from stomach of shrimp A
1450
1272
1315
1410
1348
1426
1534
1652
1677
1807
1938
1947
1968
1246
2016
l-HMC D3 cDNA from heaptopancreas of shrimp A

C

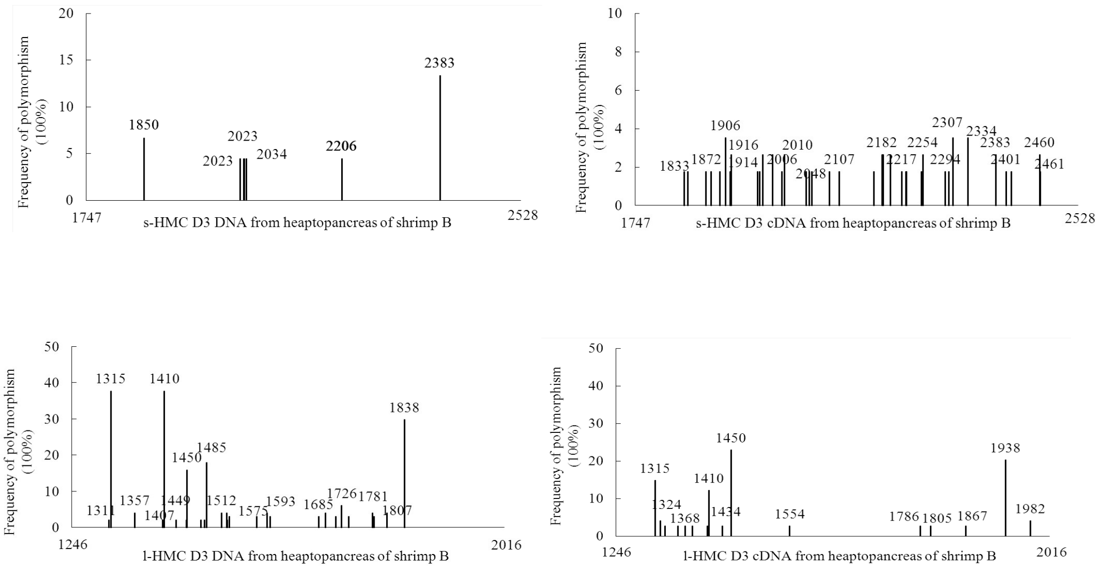
Frequency of polymorphism (100%)
s-HMC D3 DNA from heaptopancreas of shrimp B
1850
2023
2023
2034
2206
2383
s-HMC D3 cDNA from heaptopancreas of shrimp B
l-HMC D3 DNA from heaptopancreas of shrimp B
1315
1410
1450
1485
1838
l-HMC D3 cDNA from heaptopancreas of shrimp B
1315
1450
1410
1938
1554
1786
1805
1867
1982

D

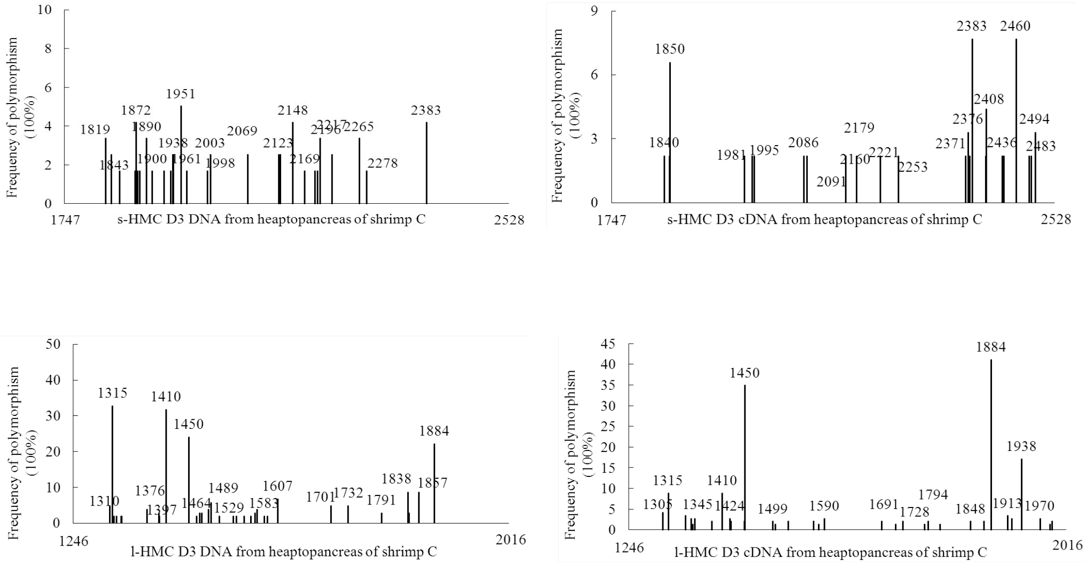
Frequency of polymorphism (100%)
s-HMC D3 DNA from heaptopancreas of shrimp C
1951
2148
2383
s-HMC D3 cDNA from heaptopancreas of shrimp C
1850
2383
2460
l-HMC D3 DNA from heaptopancreas of shrimp C
1315
1410
1450
1884
l-HMC D3 cDNA from heaptopancreas of shrimp C
1450
1884
1938

E

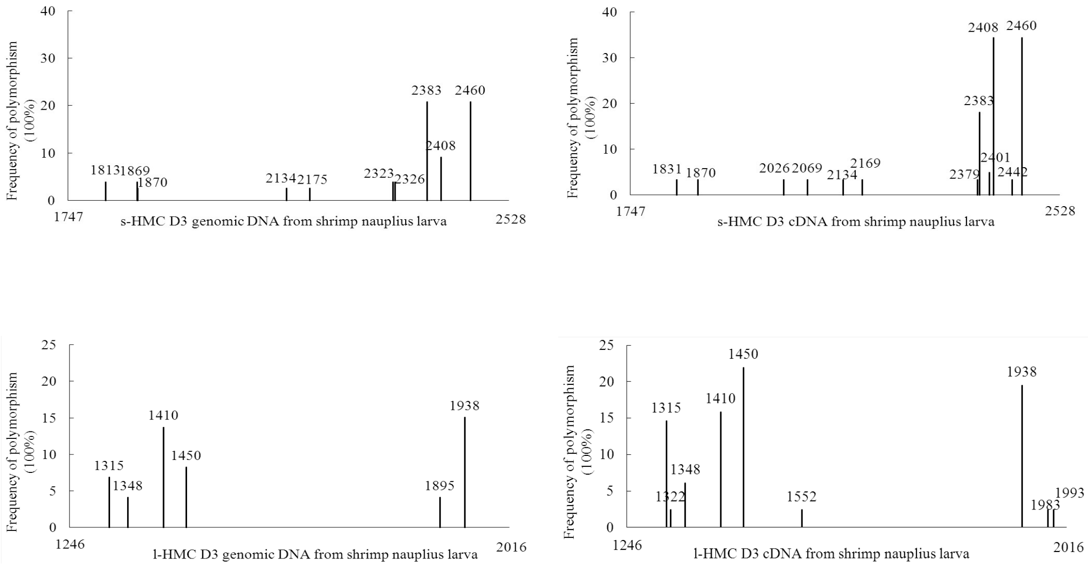

Figure S3. SNP distributions and frequencies of polymorphism (%) of HMC D3 region from shrimp *Litopenaeus vanammei*. (**A**) S-HMC D3 sequences of shrimp A genomic DNA and cDNA from heart, gill, stomach and heaptopancreas, respectively. (**B**) L-HMC D3 sequences of shrimp A genomic DNA and cDNA from heart, gill, stomach and heaptopancreas, respectively. (**C**) S-HMC D3 and L-HMC D3 sequences of shrimp B genomic DNA and cDNA, respectively. (**D**) S-HMC D3 and L-HMC D3 sequences of shrimp C genomic DNA and cDNA, respectively. (**E**) S-HMC D3 and L-HMC D3 sequences of shrimp nauplius larva genomic DNA and cDNA, respectively.
