# Supplementary material for: C-Terminal Domain of Hemocyanin, a Major Antimicrobial Protein from Litopenaeus vannamei: Structural Homology with Immunoglobulins and Molecular Diversity
Source: Front Immunol. 2017 Jun 13;8:611. doi: 10.3389/fimmu.2017.00611 (PMC5468459; doi:10.3389/fimmu.2017.00611)
Supplement: Supplementary file 4 [file table_1.pdf]

Table S1A Tores-Taylor-Thornon distances of every two HMC sequences in three

Sequence to sequence	D1	D2	D3
AJ344361-AJ516004	0.015745	0.007777	0.015846
AJ344361-AJ272095	0.392584	0.194723	0.637696
AJ516004-AJ272095	0.388918	0.203756	0.617277
AJ344361-AY193781	0.440192	0.181372	0.632863
AJ516004-AY193781	0.421422	0.190386	0.630856
AJ272095-AY193781	0.24749	0.130577	0.495439
AJ344361-AJ937836	0.468375	0.313171	0.735327
AJ516004-AJ937836	0.464096	0.323006	0.726007
AJ272095-AJ937836	0.379486	0.234769	0.784903
AY193781-AJ937836	0.422229	0.240327	0.733223
AJ344361-X82502	0.556569	0.307187	0.808822
AJ516004-X82502	0.560179	0.316933	0.800151
AJ272095-X82502	0.656761	0.248381	0.773401
AY193781-X82502	0.644245	0.220204	0.80193
AJ937836-X82502	0.654625	0.280712	0.807987
Mean	0.447528	0.226219	0.666782

Table S1B Nonsynonymous substitution rates (Ka) of every

Sequence to sequence	D1	D2	D3
AJ344361-AJ516004	0.00747	0.00384	0.00716
AY193781-AJ344361	0.24472	0.09727	0.32517
AJ272095-AJ344361	0.21873	0.09608	0.30907
AJ344361-X82502	0.29409	0.17281	0.42997
AJ937836-AJ344361	0.29518	0.18139	0.39892
AY193781-AJ516004	0.24169	0.10171	0.32477
AJ272095-AJ516004	0.21796	0.10051	0.30227
AJ516004-X82502	0.29744	0.17763	0.42181
AJ937836-AJ516004	0.29773	0.18637	0.39777
AJ272095-AY193781	0.13802	0.0744	0.27124
AY193781-X82502	0.32702	0.1289	0.4338
AY193781-AJ937836	0.24769	0.14053	0.39875
AJ272095-X82502	0.34185	0.14677	0.39721
AJ272095-AJ937836	0.21498	0.13864	0.45793
AJ937836-X82502	0.38133	0.16941	0.44348
Mean	0.25106	0.12775	0.35462

Table S1C Score values and E values in the alignments of hemocyanin D3 with human Ig

Query sequence	Subject sequence	Score	E value
Immunoglobulin kappa chain (gi|722524) [*Homo sapiens*]	Hemocyanin (gi|854403) [*Penaeus vannamei*]	27	3e-05
	Hemocyanin (gi|7414468) [*Litopenaeus vannamei*]	26	7e-05
	Hemocyanin (gi|16612121) [*Penaeus monodon*]	26	7e-05
Hemocyanin(gi|7414468) [*Litopenaeus vannamei*]	Immunoglobulin light chain variable region(gi|22095259) [*Homo sapiens*]	22	2e-04
	Immunoglobulin lambda light chain variable region(gi|3143574) [*Homo sapiens*]	22	2e-04
	Immunoglobulin kappa chain variable region (gi|722486) [*Homo sapiens*]	21	2e-04
