# Supplementary material for: C-Terminal Domain of Hemocyanin, a Major Antimicrobial Protein from Litopenaeus vannamei: Structural Homology with Immunoglobulins and Molecular Diversity
Source: Front Immunol. 2017 Jun 13;8:611. doi: 10.3389/fimmu.2017.00611 (PMC5468459; doi:10.3389/fimmu.2017.00611)
Supplement: Supplementary file 5 [file table_2.pdf]

Table S2 *E. coli* outer membrane proteins that bound to hemocyanine

Accession name	Character description	Subcellular location	MW/PI	Peptides matched	Sequence coverage (%)	Moscow score
OMPT_ECOLI	Protease	OM	35540 /5.8	6	25	72
OMPC_ECOLI	Porin	OM	40343/4.58	8	26	125
FADL_ECOLI	long-chain fatty acid transport protein	OM	48742/ 5.09	8	28	68
OMPW_ECOLI	a receptor for colicin S4	OM	22928/6.03	4	38	56
OmpX_ECOLI	integral OMP	OM	16350/5.04	8	49	122
OMPA_ECOL	porin	OM	37201/5.99	6	28	85
OMPA_ECOL	porin	OM	37201/5.99	5	26	73
OMPA_ECOL	porin	OM	37201/5.99	8	32	107

OM, outer membrane
